# MAnorm: a robust model for quantitative comparison of ChIP-Seq data sets

**DOI:** 10.1186/gb-2012-13-3-r16

**Published:** 2012-03-16

**Authors:** Zhen Shao, Yijing Zhang, Guo-Cheng Yuan, Stuart H Orkin, David J Waxman

**Affiliations:** 1 Departments of Pediatric Oncology and Computational Biology, Dana-Farber Cancer Institute, 44 Binney Street, Boston, MA 02115, USA; 2 Division of Pediatric Hematology-Oncology, The Karp Family Research Laboratories, Children's Hospital, 300 Longwood Ave, Boston, MA 02115, USA; 3 Division of Cell and Molecular Biology, Department of Biology, Boston University, 5 Cummington Street, Boston, MA 02215, USA; 4 Harvard Stem Cell Institute and the Howard Hughes Medical Institute, 1 Blackfan Circle, Karp Research Building, Children's Hospital, Boston, MA 02115, USA

## Abstract

ChIP-Seq is widely used to characterize genome-wide binding patterns of transcription factors and other chromatin-associated proteins. Although comparison of ChIP-Seq data sets is critical for understanding cell type-dependent and cell state-specific binding, and thus the study of cell-specific gene regulation, few quantitative approaches have been developed. Here, we present a simple and effective method, MAnorm, for quantitative comparison of ChIP-Seq data sets describing transcription factor binding sites and epigenetic modifications. The quantitative binding differences inferred by MAnorm showed strong correlation with both the changes in expression of target genes and the binding of cell type-specific regulators.

## Background

Chromatin immunoprecipitation followed by massively parallel DNA sequencing (ChIP-Seq) has become the preferred method to determine genome-wide binding patterns of transcription factors and other chromatin-associated proteins [1]. With the rapid accumulation of ChIP-Seq data, comparison of multiple ChIP-Seq data sets is increasingly becoming critical for addressing important biological questions. For example, comparison of biological replicates is commonly used to find robust binding sites, and the identification of sites that are differentially bound by chromatin-associated proteins in different cellular contexts is important for elucidating underlying mechanisms of cell type-specific regulation. Although ChIP-Seq data generally exhibit high signal-to-background noise (S/N) ratios compared to ChIP-on-chip datasets, there are still significant challenges in data analysis due to variation in sample preparation and errors introduced in sequencing [1].

Several methods have been proposed for finding ChIP-enriched regions in a ChIP-Seq sample compared to a suitable negative control (for example, mock or non-specific immunoprecipitation). These involve fitting a model derived from negative control and/or sample low read intensity (background) regions, and then applying this model to identify ChIP-enriched regions (peaks) [2-4]. However, few methods have been proposed for comparison of ChIP-Seq samples. The simplest approach classifies the peaks from each sample as either common or unique, based on whether or not the peak overlaps with peaks in other samples [5-10]. Although this method can identify general relationships between peak sets from different samples, the results are highly dependent on the cutoff used in peak calling, which is difficult to select in a completely objective manner. Moreover, common peaks may show differential binding between the samples being compared, while other peaks may be identified as unique to one sample simply because they fall below an arbitrary cutoff in the other sample. Differences in background levels further confound analysis. Consequently, quantitative comparison of ChIP-Seq samples, while important for extracting maximal biological information, is fraught with numerous challenges.

An intuitive and widely used approach of quantitative comparison relies on rescaling data on the basis of the total number of sequence reads. However, this method is inadequate and may introduce errors when the S/N ratio varies between samples. Recently, statistical tools have been developed to discover regions that exhibit significant differences between two ChIP-Seq data sets. For example, *Xu et al. *[11] proposed a hidden Markov model-based method to detect broad chromatin domains associated with distinct levels of histone modifications between two cell types. Other peak calling programs identify differential binding regions between two ChIP-Seq data sets by using one data set as sample and the other as control [2-4]. Since these methods also rely on the total number of reads (or background region reads) to re-scale the data, they fail to circumvent problems associated with different S/N ratios. In an alternative approach, Taslim *et al. *[12] proposed a nonlinear method that uses locally weighted regression (LOWESS) for ChIP-Seq data normalization. The underlying assumption of this method is that the genome-wide distribution of read densities has equal mean value and variance across samples [12]. A potential problem with this approach is that global symmetry will be introduced after normalization, an assumption that may not be valid when comparing biological samples with different numbers of binding sites. In addition, this method normalizes samples based on the absolute difference of read counts instead of log_2 _ratio commonly used in traditional MA plot methods [13], and thus the differences deduced by this method cannot be used directly for quantitative comparison with other observations of biological significance, such as fold changes in gene expression.

Here, we describe a simple and effective model, termed MAnorm, to quantitatively compare ChIP-Seq data sets. To circumvent the issue of differences in S/N ratio between samples, we focused on ChIP-enriched regions (peaks), and introduced a novel idea, that ChIP-Seq common peaks could serve as a reference to build the rescaling model for normalization. This approach is based on the empirical assumption that if a chromatin-associated protein has a substantial number of peaks shared in two conditions, the binding at these common regions will tend to be determined by similar mechanisms, and thus should exhibit similar global binding intensities across samples. This idea is further supported by motif analysis that we present. MAnorm exhibits good performance when applied to ChIP-Seq data for both epigenetic modifications and transcription factor binding site identification. Importantly, quantitative differences inferred by MAnorm are strongly correlated with differential expression of target genes and the binding of cell type-specific regulators. Comparisons to prior methods using genome-wide signals for normalization reveal that MAnorm is free of bias and better reflects authentic biological changes. Therefore, MAnorm should serve as a powerful tool in probing mechanisms of gene regulation.

## Results

### Model description

**Figure 1:**
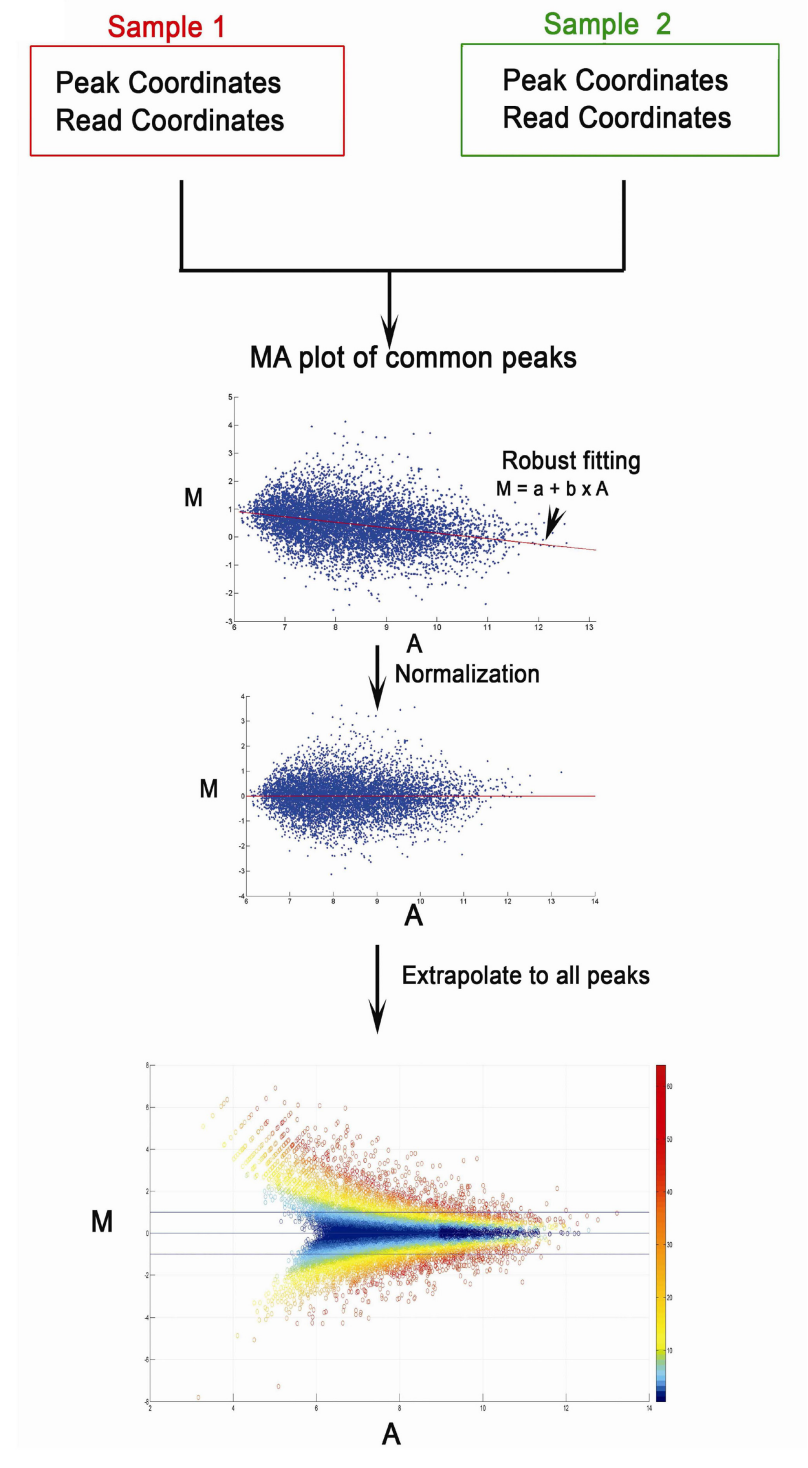
**Workflow of MAnorm**. MAnorm takes the coordinate of all peaks and aligned reads in both samples as input. The (*M, A*) value of each common peak is then calculated and plotted, where *M *= log_2 _(Read density in sample 1/Read density in sample 2) and *A *= *0.5 *× log_2 _(Read density in sample 1 × Read density in sample 2). Robust regression is subsequently applied to the (*M, A*) values of all common peaks and a linear model is derived. Finally, the linear model is extrapolated to all peaks for normalization. A *P*-value is also calculated for each peak to describe the statistical significance of read intensity difference between the two samples being compared.

Data normalization is an important step in sequencing data analysis. However, normalization of ChIP-Seq data is a difficult task due to the differential S/N ratio across samples (see Discussion). These differences cannot simply be addressed using traditional microarray data normalization methods, such as quantile normalization [14] and MA plot followed by LOWESS regression [13]. Here we borrow the idea of the MA plot and propose a novel method for quantitative comparison of ChIP-Seq data sets based on two empirical assumptions. First, we assume the true intensities of most common peaks are the same between two ChIP-Seq samples. This assumption is valid when the binding regions represented by the common peaks show a much higher level of co-localization between samples than that expected at random, and thus binding at the common peaks should be determined by similar mechanisms and exhibit similar global binding intensity between samples. Second, the observed differences in sequence read density in common peaks are presumed to reflect the scaling relationship of ChIP-Seq signals between two samples, which can thus be applied to all peaks. Based on these hypotheses, the log_2 _ratio of read density between two samples (*M*) was plotted against the average log_2 _read density (*A*) for all peaks, and robust linear regression was applied to fit the global dependence between the *M-A *values of common peaks. Finally, the derived linear model was used as a reference for normalization and extrapolated to all peaks. The normalized *M *value was then used as a quantitative measure of differential binding in each peak region between two samples, with peak regions associated with larger absolute *M *values exhibiting greater differences in binding. The workflow of the method, MAnorm, is shown in Figure 1. The MAnorm package is available for download in Additional file 1.

### Comparison of cell line-dependent epigenetic modifications using MAnorm

**Figure 2:**
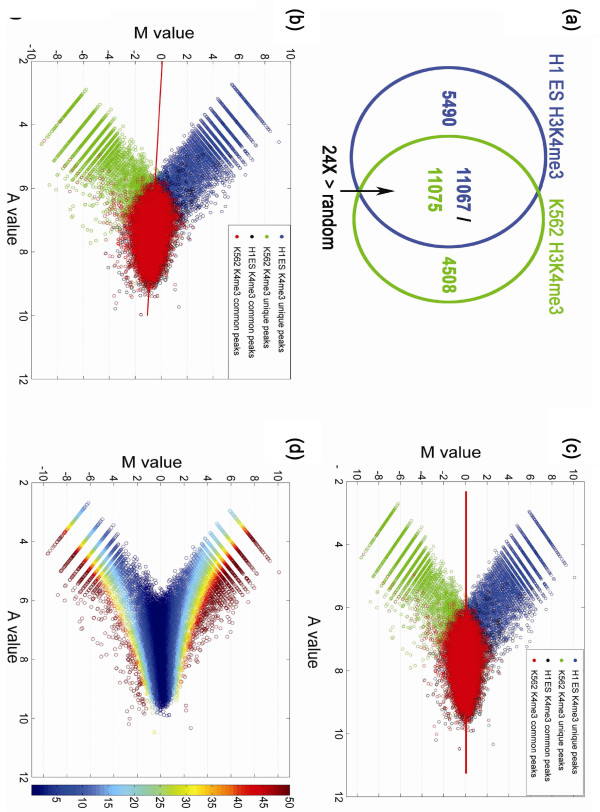
**Normalization of H3K4me3 ChIP-Seq data in H1 ES cells and K562 cells. (a) **Venn diagram representing the overlap of H3K4me3 peaks between H1 ES and K562 cells. The overlap of peaks between the two cell lines was 24-fold greater than that observed for random permutations of the peak sets. **(b,c) **MA plots of all peaks from both samples before (b) and after MAnorm (c). The red line is the linear model derived from common peaks by robust regression. Purple and green circles represent unique peaks; red and black circles represent common peaks. **(d) ***P*-values associated with normalized peaks, displayed as an MA plot, with the color range representing -log_10 _*P*-value. Most peaks associated with |*M*| > 1 have a *P*-value < 10^-10^.

Differential epigenetic modifications are closely associated with many developmental and disease processes [15]. As such, quantitative comparison of ChIP-Seq signals across multiple cell types may help elucidate underlying epigenetic mechanisms of disease and tissue-specific regulation. We applied MAnorm to analyze the differences between H1 human embryonic stem (ES) cells and two disease-related cell lines, K562 and HeLaS3, for two histone modifications positively associated with gene expression, H3K4me3 and H3K27ac. For each chromatin mark, peaks identified in each cell line showed substantial overlap with those from the other two cell lines, with the overlap ranging from 16- to 24-fold greater than the overlap observed by random permutations (Figure 2a; Supplementary Figure 1 in Additional file 2). Before normalization, the MA plots exhibited an overall global dependence of *M *value on *A*, which was closely fitted by a linear model derived by robust regression (Figure 2b; Supplementary Figure 2 in Additional file 2). A similar global dependence was evident in comparisons of biological replicates (Supplementary Figure 7 in Additional file 2; discussed below), indicating the dependence of *M *on *A *does not reflect biological changes but is due mainly to systemic bias and noise. After application of MAnorm to remove this dependence from the set of common peaks, the distribution of common peaks became highly symmetric with respect to the new *A *axis. Furthermore, the two sets of unique peaks became more symmetric in all comparisons (Figure 2c; Supplementary Figure 2 in Additional file 2). These observations suggest that the ChIP-Seq signals in all peaks follow a similar scaling relationship and that the extrapolation of the linear model from common peaks to all peaks is valid. The significance of differential binding in each peak region was determined using a *P*-value calculated based on a Bayesian model developed by Audic and Claverie [16] (Figure 2d; Supplementary Figure 2 in Additional file 2).

**Figure 3:**
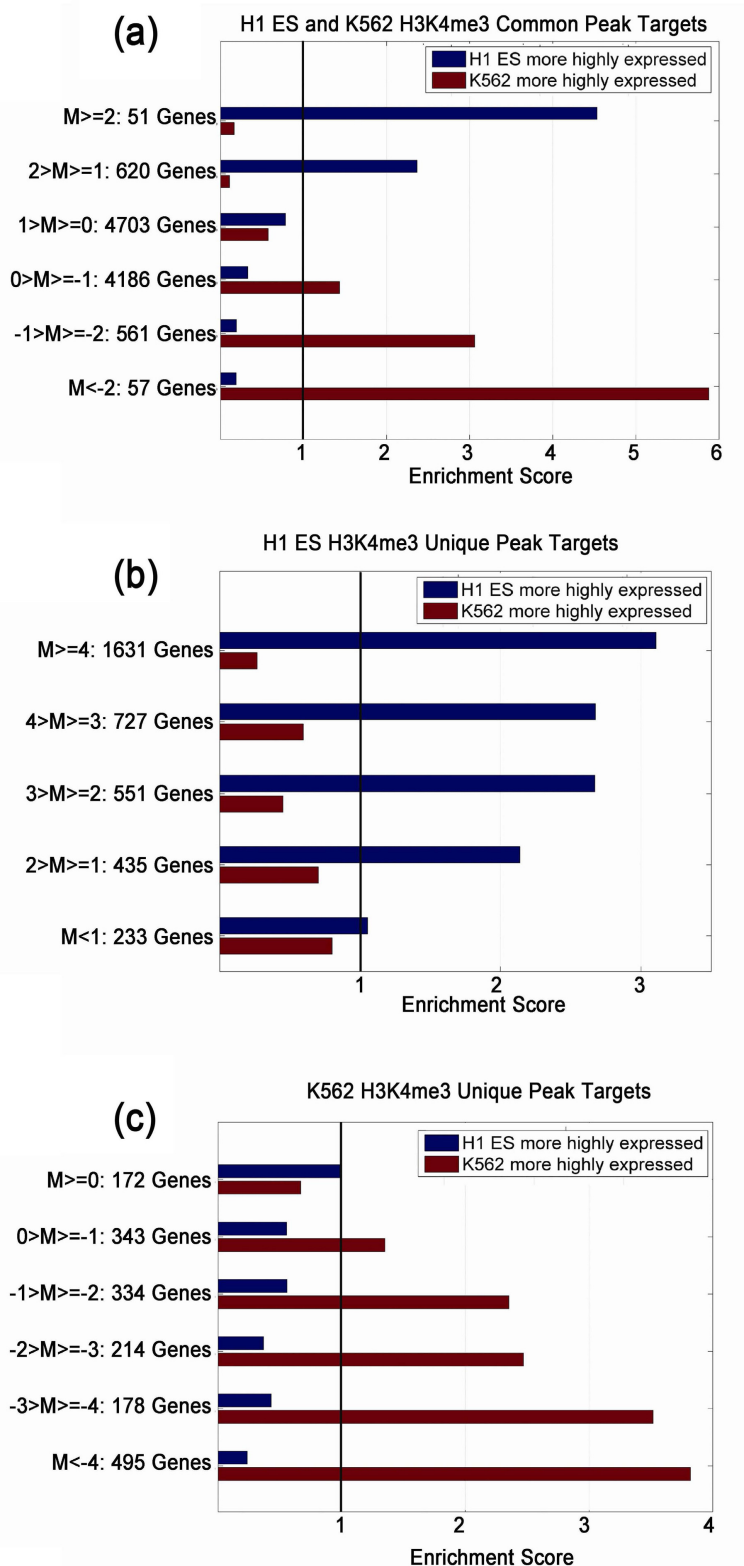
**Quantitative differences in H3K4me3 marks between two cell lines are strongly correlated with cell type-specific expression of peak targets. (a) **Enrichment of the target genes of all common H3K4me3 peaks in H1 ES cells and K562 cells in cell type-specifically expressed genes as identified by SAM (see Materials and methods). The target genes were grouped by the *M *values of nearby peaks and the enrichment scores were calculated as the ratio of overlap between target genes grouped by *M *value and differentially expressed genes compared to expected overlap at random. **(b,c) **Enrichment of the the target genes of all unique H3K4me3 peaks in H1 ES cells (b) or K562 cells (c) in cell type-specifically expressed genes.

Next, we investigated the relationship between the *M *value (= *log_2 _*(Read density in cell type 1/Read density in cell type 2)) and the change in expression of peak targets between cell types. In general, target genes associated with positive *M *values - that is, peaks with higher H3K4me3 and H3K27ac read intensity in cell type 1 - were enriched in genes more highly expressed in cell type 1. Conversely, target genes associated with negative *M *values were enriched in genes more highly expressed in cell type 2 (Figure 3; Supplementary Figure 3 in Additional file 2). These findings are consistent with the activating role of these two histone modifications [17]. Notably, the enrichment score of genes more highly expressed in cell type 1 showed strong positive correlation with the *M *values, while the enrichment score of genes more highly expressed in cell type 2 correlated negatively with *M*, suggesting that the *M *statistics determined by MAnorm serve as an indicator of cell type-specificity for the epigenetic marks in peak regions (Figure 3; Supplementary Figure 3 in Additional file 2). Furthermore, the target genes associated with an absolute *M *value > 1 were significantly enriched in genes highly expressed in the corresponding cell type among all our comparisons, implying that the absolute *M *value of 1 is a suitable cutoff for defining cell type-specifically marked genes. It should be noted that many common target genes were associated with *M *values far from 0, and were still highly enriched for cell type-specifically expressed genes (Figure 3a; Supplementary Figure 3a in Additional file 2), indicating that the differential epigenetic marks at these genes are also functional. On the other hand, those unique target genes with *M *values near zero displayed much weaker enrichment of cell type-specifically expressed genes (Figure 3b, c; Supplementary Figure 3b, c in Additional file 2), indicating that they are not uniquely marked in one cell type. MAnorm also exhibited good performance when applied to ChIP-seq datasets composed of broad, diffuse peaks, such as histone modifications like H3K36me3 (Supplementary Figure 4 in Additional file 2 and Supplementary Text in Additional file 3). In conclusion, MAnorm quantitatively describes authentic binding differences of chromatin-associated proteins, and thus represents an improvement over arbitrary definitions of common and unique targets based on peak overlap between samples.

**Figure 4:**
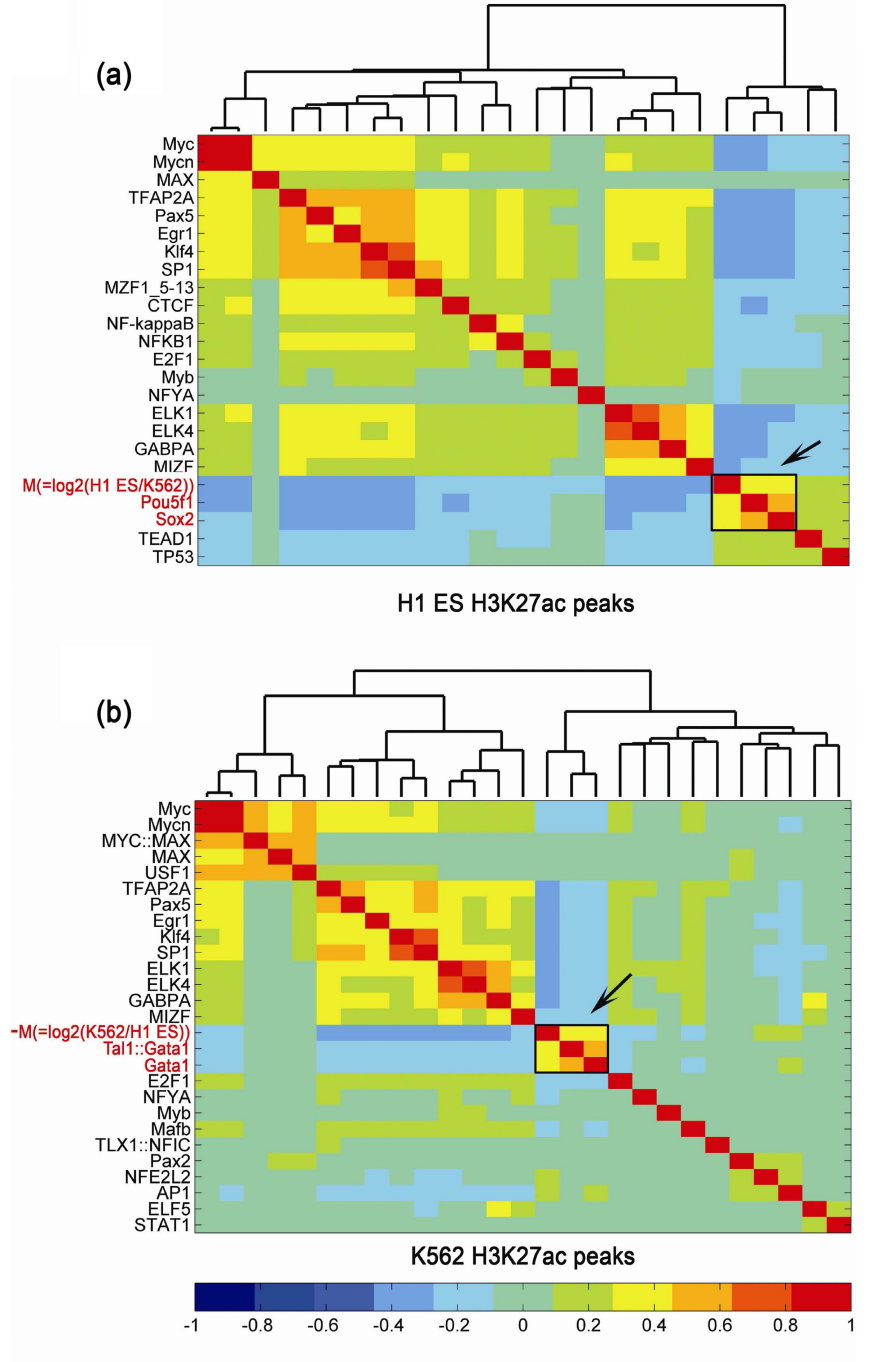
**Hierarchical clustering of the *M *value and motif scores in all H3K27ac peaks of H1 ES cells and K562 cells. (a,b) **Hierarchical clustering was applied to the correlation coefficients of *M *values (= log2 (Read density in H1 ES/Read density in K562)) or -*M *values (= log2 (Read density in K562/Read density in H1 ES)) of all H3K27ac peaks identified in H1 ES cells (a) or K562 cells (b), with motif scores determined for 130 JASPAR vertebrate core motifs in the peak regions. Only the motifs significantly enriched in the peaks of either cell type are shown here (enrichment score > 1.2 and Bonferroni corrected *P*-value < 1e-5 by Fisher exact test). The names of the motifs closely clustered with *M *value or -*M *value are colored in red.

### Identification of cell type-specific regulators directly associated with differential binding

A conventional strategy to identify cell type-specific regulators associated with changes in epigenetic marks relies on the identification of transcription factor binding sites that are highly enriched in unique peak regions. This method often yields multiple candidates, and thus complicates the identification of key regulators associated with the differences in epigenetic marks in each cell type. One advantage of the continuous *M *value determined by MAnorm is that it can be used to identify potential regulators driving cell type-specific epigenetic modifications. To do so, we searched for motifs that show the highest correlation with *M *values for all peaks. For example, we compared H1 ES and K562 cell lines for differences in H3K27ac, a histone mark that serves as an indicator of both active promoters and cell type-specific enhancers [18,19]. We found that OCT4 (POU5F1) and SOX2 binding motifs were closely clustered with the *M *value (= log_2 _(H3K27ac read density in H1 ES cells/H3K27ac read density in K562 cells) of H3K27ac peaks (Figure 4a), suggesting the corresponding factors are closely related to the activation of ES cell-specific genes and *cis*-elements. In contrast, -*M *value (= log_2 _(H3K27ac read density in K562 cells/H3K27ac read density in H1 ES cells) formed a compact module with the binding motifs for transcription factors GATA1 and SCL (TAL1) (Figure 4b), suggesting their roles as regulators favoring H3K27ac modification in K562 cells. These findings are consistent with the established roles of OCT4-SOX2 in ES cell self-renewal [20,21] and GATA-SCL in hematopoiesis and leukemogenesis [5]. On the other hand, several motifs, including MYC and ETS motifs (for example, ELK1, ELK4, GABPA), were highly enriched in both peak sets, but showed no association with the differential binding of H3K27ac (specifically, *M *value); this indicates they are involved in H3K27ac modification in a non-cell type-specific manner. This finding in turn supports the working assumption of our model that binding at most common peaks is determined by similar mechanisms. Furthermore, upon comparison of the H3K27ac marks in H1 ES or K562 cells with those in HeLaS3 cells, these same transcription factor motifs were tightly associated with the H1 ES or K562-specific enrichment of H3K27ac marks at the corresponding target regions (Supplementary Figure 5 in Additional file 2), indicating the clustering results are robust. Thus, MAnorm serves as a powerful tool to uncover transcription factor motifs and factors critical for cell-specific gene regulation.

**Figure 5:**
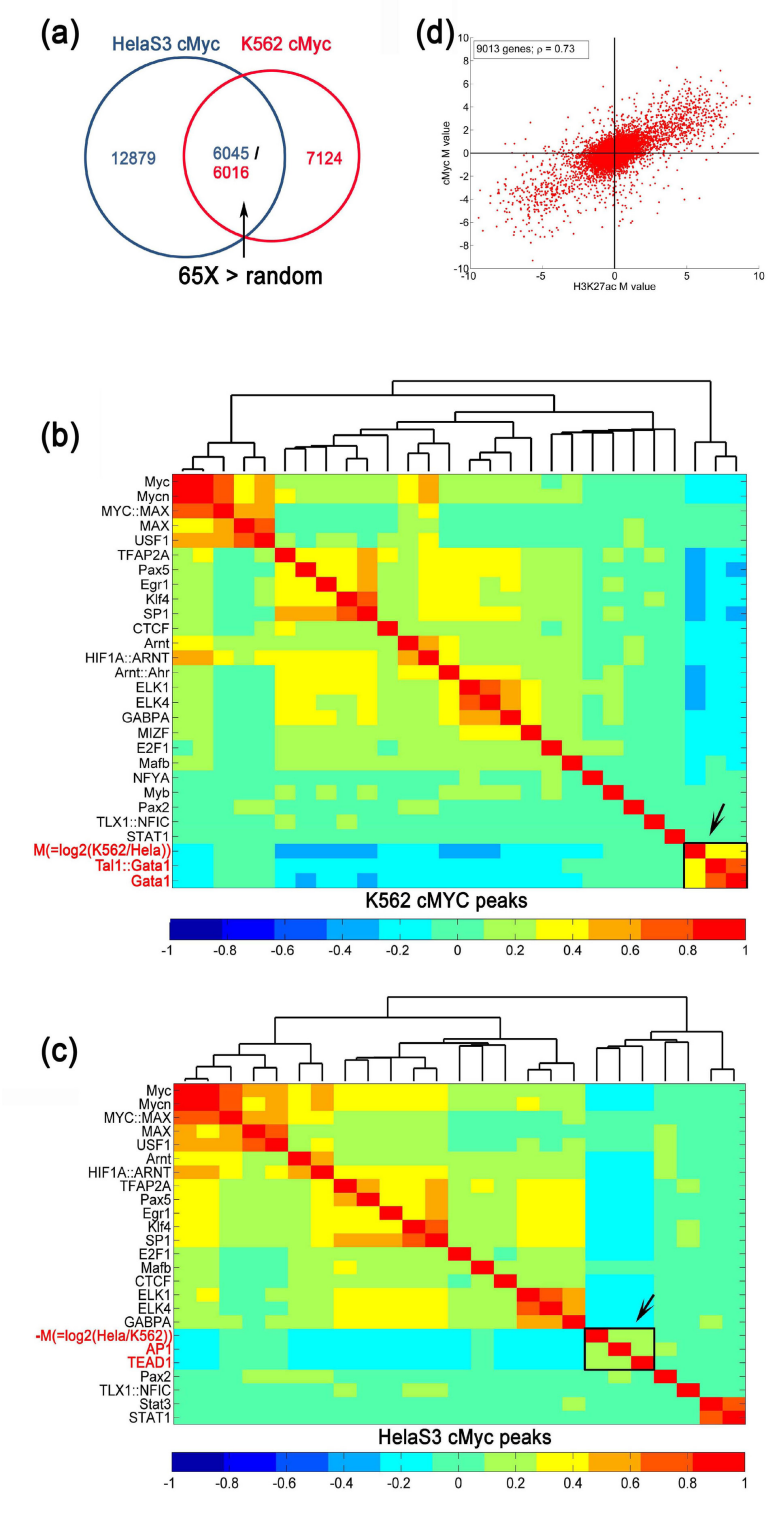
**Comparison of c-Myc ChIP-Seq data between HeLaS3 and K562 cell lines. (a) **Venn diagram showing the overlap of c-Myc binding site peaks between HeLaS3 and K562 cell lines. The overlap of cMyc peaks between the two cell lines was 65-fold greater than that observed for random permutations of the peak sets. **(b,c) **Hierarchical clustering of correlation coefficients of *M *value or -*M *value of all c-Myc peaks in HeLaS3 cells (b) and K562 cells (c) with the motif scores in the corresponding peak regions. Only significantly enriched motifs are shown. **(d) **Scatter plot of the *M *values determined for c-Myc binding versus the *M *values for H3K27ac based on ChIP-Seq comparisons between HeLaS3 and K562 cell lines.

### Differences in c-Myc binding between HeLaS3 and K562 cells

The oncogene Myc (c-Myc) is an important transcriptional regulator in both ES cells and cancer cells [22,23]. Mechanisms underlying its cell type-specific binding are largely unknown. We applied MAnorm to quantify differential binding of c-Myc in HeLaS3 and K562 cells and explored its relationship with other factors. Using a *P*-value cutoff of 1E-6, 18,924 peaks were detected in the c-Myc ChIP-Seq data set of HeLaS3 cells, and 13,140 peaks were detected in K562 cells; approximately 6,000 peaks were common to both cell lines (Figure 5a). MAnorm largely removed the global dependence of M on A (Supplementary Figure 6a, b in Additional file 2). A significant fraction of c-Myc peaks were associated with *M *values far from zero, suggesting that c-Myc has a large number of differential binding loci between HeLaS3 cells and K562 cells. To search for cell line-specific co-factors that might contribute to such differential binding, we performed hierarchical clustering between the *M *statistics inferred by MAnorm and the motif scores in the c-Myc binding peaks. The c-Myc motif was highly enriched in both sets of c-Myc peaks (data not shown), but did not show significant correlation with *M *statistics in either clustering map (Figure 5b, c), indicating that the c-Myc motif is not responsible for the cell line-differential binding seen for c-Myc. Of note, the *M *statistic (= log_2 _(c-Myc read density in K562/c-Myc read density in HeLaS3)) clustered with the motifs of two other factors, GATA1 and SCL (TAL1) (Figure 5b), and the -*M *statistic (= log_2 _(c-Myc read density in HeLaS3/c-Myc read density in K562) clustered with the motifs of AP1 and TEAD1 (Figure 5c). Strikingly, these clustering patterns were highly similar to those obtained from the comparison of the H3K27ac mark between these two cell types (Supplementary Figure 5b in Additional file 2), suggesting an underlying correlation between the cell type-specific binding of c-Myc and the H3K27ac mark. To test whether this was the case, we mapped c-Myc binding sites to gene promoters, and found that for the 9,013 genes targeted by both c-Myc and H3K27ac, the Pearson correlation coefficient between the *M *statistics of c-Myc and H3K27ac was 0.73 (Figure 5d), lending further support to our clustering result.

**Figure 6:**
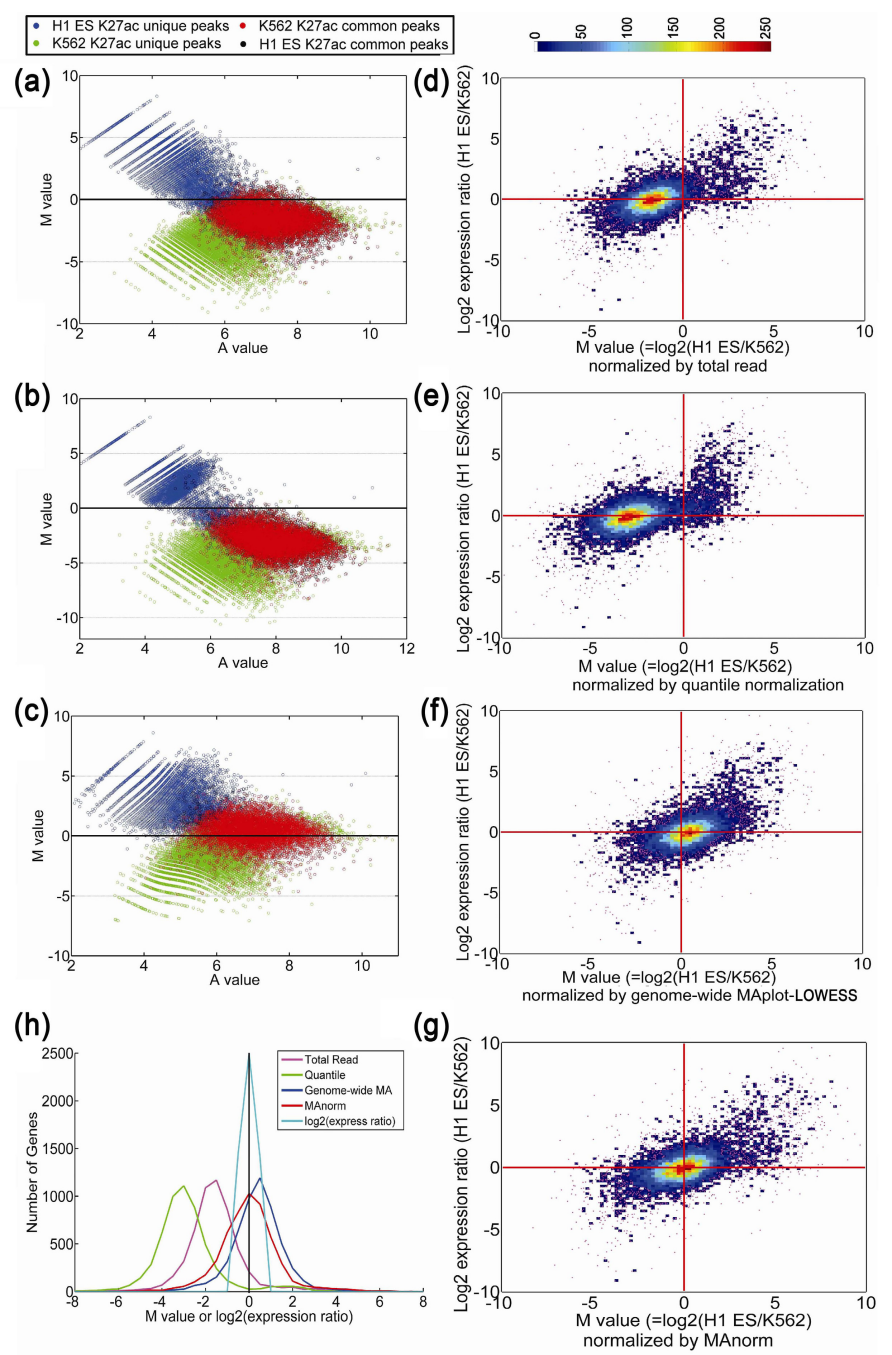
**Comparison of different normalization models. (a-c) **MA plot of H3K27ac peaks in H1 ES cells and K562 cells after normalization by total reads (a), quantile normalization (b) and genome-wide MA plot followed by LOWESS regression (c). The corresponding MA plot based on MAnorm is shown in Supplementary Figure 2a in Additional file 2. **(d-g) **Scatter plot of log2 expression ratios of target genes between H1 ES cells and K562 cells versus the *M *values normalized by total reads (d), quantile normalization (e), genome-wide MA plot followed by LOWESS normalization (f), and MAnorm (g). The color bar represents the density of dots in the scatter plot and purple dots represent the outliers separated from the others. **(h) **Distribution of *M *values for each normalization method and distribution of log2 expression ratios of non-differentially expressed target genes (fold-change < 1.5). T-statistics and *P*-values calculated based on one sample Students' *t*-test comparing to 0 for each normalization method were as follows: MAnorm, t-statistic = -0.55 and *P *= 0.58 by *t*-test; total reads normalization, t-statistic = -88 and *P *< 1E-100; quantile normalization, t-statistic = -140 and *P *< 1E-100; genome-wide MA, t-statistic = 24 and *P *< 1E-100. For non-differentially expressed target genes, t-statistic = -0.76 and *P *= 0.45.

### Application to the integration of ChIP-Seq replicates

Integrating ChIP-Seq data from multiple biological replicates, which in some cases are generated by different laboratories and/or using different platforms, may be employed to reduce the false positive rate in identified binding sites. A simple approach is to define a stringent set of peaks composed only of the common peaks shared by two or more replicates. However, this method is highly sensitive to peak cutoff and may exclude peaks that have similar ChIP intensities between replicates. Moreover, some common peaks that show dramatic differences in read density are retained. Therefore, to make full use of the information in biological replicates, a quantitative comparison of peak intensity is particularly useful. We have applied MAnorm to compare two replicates of H1 ES cell H3K27ac ChIP-Seq data. After application of MAnorm (Supplementary Figure 7a, b in Additional file 2), many of the unique peaks were associated with *M *values close to zero, indicating that these peaks exhibit good reproducibility between replicates. On the other hand, there remained a small fraction of common peaks with *M *values far from zero, representing strong signal differences between replicates. Next, we showed that the *M *value between replicates is a good indicator of H3K27ac target gene expression. We grouped H3K27ac target genes by the absolute value of *M *statistics and calculated the expression distribution of each gene group. Given that H3K27ac marks are positively associated with gene expression, we anticipated that more highly expressed genes will have stronger H3K27ac marks, and therefore be more reliable. In fact, we observed that genes having higher expression tend to be the targets of H3K27ac peaks with lower absolute *M *values, that is, peaks showing smaller difference between replicates, for both common peaks and unique peaks (Supplementary Figure 7c-e in Additional file 2). Furthermore, by overlapping the above set of ENCODE peaks with H3K27ac peaks for H1 ES cells generated in a different laboratory [19], we found that a much lower proportion of the peaks with |*M*| > 1 were covered by the new peak set than those with |*M*| < 1 (Supplementary Figure 7f in Additional file 2). This suggests that |*M*| = 1 can also be used as an empirical cutoff to filter unreliable peaks. Thus, MAnorm can be used both to check whether two replicates are concordant, and also to obtain high confidence peak lists by filtering out inconsistent peaks. Compared with arbitrary removal of unique peaks, MAnorm allows for better use of replicate peak data. The MAnorm package (Additional file 1) provides the opportunity to list concordant and non-concordant peaks between two samples based on user-specified cutoffs, with the concordant peak list corresponding to high-confidence peaks.

### Comparison with other methods

We compared the performance of MAnorm with three widely used normalization methods that use genome-wide signals as reference, namely, normalization by total reads, quantile normalization, which assumes the genome-wide distribution of read densities to be the same across samples, and normalization using a genome-wide MA plot followed by LOWESS regression. We used all four methods to compare H3K27ac ChIP-Seq data between H1 ES and K562 cells. The MA plot normalized by MAnorm (Supplementary Figure 2a in Additional file 2) was relatively symmetric, while corresponding plots obtained by the other three normalization methods remained highly asymmetric. Of note, the common peaks showed a clear global bias towards stronger binding in K562 cells for total read normalization and quantile normalization (Figure 6a, b) and toward H1 ES cells for genome-wide MA plot normalization (Figure 6c). To examine which method better reflects a true biological signal, we compared *M *values normalized by all four methods with the expression change of target genes. If a specific type of histone modification is closely related to gene regulation, the direction of histone modification change should be consistent with that of the change in expression of the target genes. By visual inspection, we found this was true for the *M *values normalized by MAnorm (Figure 6g). In contrast, *M *values normalized by the other three methods were inconsistent with the log2-expression ratios of target genes (Figure 6d-f). Specifically, most of the genes with no change in H3K27ac levels (*M *= 0) had higher (total read and quantile normalization) or lower (genome-wide MA plot normalization) expression in H1 ES cells compared to K562 cells; while the majority of the genes expressed at similar levels in these two cell types were associated with negative (total read and quantile normalization) or positive (genome-wide MA plot normalization) *M *values, that is, they had higher (total read and quantile normalization) or lower (genome-wide MA plot normalization) levels of H3K27ac in K562 cells.

To quantitatively measure the bias of the *M *values given by the above normalization methods, we first collected non-differentially expressed genes (fold-change < 1.5) between H1 ES cells and K562 cells. As shown in Figure 6h, these genes are indeed not differentially expressed (t-statistics = -0.76 and *P*-value = 0.45 by Students' *t*-test in comparison to an expression ratio of 1 (*M *= 0)), indicating they are suitable for our comparison. Since H3K27ac marks are closely associated with transcriptional activation, it is reasonable to assume that these non-differentially expressed genes should exhibit similar global H3K27ac levels. This is true only for H3K27ac levels determined by MAnorm, where the *M *values for H3K27ac of the non-differentially expressed target genes were not significantly different from a ratio of 1 (*M *= 0; t-statistic = -0.55 and *P*-value = 0.58 by *t*-test; Figure 6h, red curve). In contrast, *M *values for H3K27ac obtained by the other three normalization methods exhibited large deviations from *M *= 0 (t-statistic ranging from 24 to 140 and *P*-value < 1e-100; Figure 6h). Thus, MAnorm exhibits superior performance in identifying authentic biological changes.

We also compared the performance of MAnorm in detecting differential binding regions in ChIP-Seq data sets with that of two currently used statistical methods, ChIPdiff [11] and MACS [4]. For this analysis, one data set was used as sample and the other was used as control in order to detect regions with significantly elevated ChIP-Seq signals in the first data set [4]. We applied all three methods to compare ChIP-Seq data for H3K27ac marks between H1 ES cells and K562 cells (Supplementary Table 1 in Additional file 4). ChIPdiff and MACS identified four to six times more target regions associated with significantly increased ChIP-Seq signals for K562 cells compared with those found for H1 ES cells, whereas MAnorm yielded a similar number of cell type-biased peaks in each cell line. To compare the enrichment of cell type-specifically expressed genes in the sets of target genes of the differential binding regions discovered by the three methods, we selected the same number of target genes associated with top differential binding regions identified by each method. The target genes of top differential binding regions identified by MAnorm contained similar numbers of H1 ES cell highly expressed genes but a greater number of K562 cell highly expressed genes compared to those identified by ChIPdiff and MACS (Supplementary Table 1 in Additional file 4), suggesting MAnorm performs better in detecting differentially binding regions than the other two methods. Importantly, the fold changes of differential binding given by ChIPdiff and MACS were based on the total number of reads, which may not be appropriate, as discussed above. Additionally, MAnorm showed even better enrichment of cell type-specifically expressed genes in differential binding region targets than the method developed by Taslim *et al. *[12] when applied to ChIP-Seq data presented in their study (Supplementary Table 2 in Additional file 4).

## Discussion

Normalization methods are typically based on the assumption that certain properties are invariant across samples. For example, quantile normalization in gene expression microarrays renders the distribution of expression levels of all genes constant between samples [14]. Alternatively, normalization may be based on housekeeping genes, whose expression is presumed to remain constant across samples. The situation is quite different in ChIP-Seq studies, since the binding of most chromatin-associated proteins is highly dynamic and cell type-dependent. Thus, it is arbitrary to assume that the genome-wide distribution of ChIP-seq signals remains constant between samples. It is also challenging to identify reliable control genomic regions bound by a chromatin-associated protein in a non-cell type-specific manner that can serve as an internal reference for normalization. Yet another difficulty underlying ChIP-Seq studies is background noise, which is often difficult to distinguish from authentic ChIP signals. Furthermore, the S/N ratio often varies across samples. These same issues apply to DNase-Seq data sets, as discussed elsewhere [24]. In many peak-calling models, the distribution of background signal is used to normalize sample and control data, which is reasonable when control data are composed mainly of background signal, and the purpose is to identify sequence read-enriched regions within a sample that shows significant differences compared to the background. However, this approach is inappropriate for sample-to-sample comparisons, especially when the S/N difference is large across samples. For example, samples relatively free of 'noise' will yield a larger number of statistically significant peaks compared to samples with a higher level of background sequence reads, but these additional peaks may not be true cell line-specific or condition-specific peaks. In MAnorm, we focused only on regions identified as significant peaks, and thus minimized the impact of S/N differences between samples. Accordingly, the output of MAnorm focuses on peak regions most likely to be of biological relevance.

MAnorm shows improved performance when compared with other methods currently used to detect differential binding regions between ChIP-Seq data sets. More importantly, MAnorm provides a quantitative measurement of binding differences, which reflects authentic biological differences. This feature is an asset for downstream analysis, including expression assays and transcription co-factor identification studies. Although the definition of ChIP-Seq peaks is highly dependent on the cutoff used in peak calling, MAnorm is robust to cutoff selection (Supplementary Figure 8 in Additional file 2 and Additional file 3). Furthermore, the normalized read densities of each peak in both ChIP-Seq samples can be calculated from the (*M, A*) values normalized by MAnorm, and then used to evaluate whether the cutoffs used to define peaks are comparable between the ChIP-Seq samples being compared (Supplementary Figure 8 in Additional file 2 and Additional file 3).

MAnorm relies on two working assumptions. First, MAnorm is designed for quantitative comparison of ChIP-Seq data sets that have a substantial number of peak regions in common. Second, MAnorm postulates that there are no global changes in the true ChIP signals at these common peaks. We believe these underlying hypotheses are widely applicable and do not significantly restrict the use of MAnorm, as exemplified by our application of MAnorm to elucidate hormone-regulated, cell state-specific transcription factor binding in mouse liver *in vivo *[25]. For ChIP-seq samples for which there is not a significant overlap in peak sets, the binding of chromatin-associated proteins could be uncorrelated or even anti-correlated at a genome-wide scale and MAnorm would not be applicable. However, in that case a quantitative comparison would likely not be that useful. In addition, in cases where the binding patterns for a chromatin-bound factor change widely across the genome, such as following knock down of a core subunit of a chromatin-associated protein complex [26], more specific analysis would be required to quantitatively determine the global changes.

The pairwise approach to comparison of ChIP-Seq samples proposed here can be extended to multiple sample comparison, as was successfully demonstrated in the case of two-channel microarray data analysis [13]. Furthermore, it is well known that transcription factors and epigenetic modifications act together to modulate gene expression [27]. Most recently, statistical models have been developed to study such combinatorial patterns in a genome-wide fashion [28-32]. However, how changes in epigenetic marks and transcriptional factors correlate with each other across cell lines is still largely unexplored. In this study, we used MAnorm to successfully detect an underlying correlation between cell-type dependent binding of c-Myc and the H3K27ac mark in two disease-related cell types. Thus, it will be interesting to integrate quantitative changes of other epigenetic marks and transcriptional factors for further elucidation of the complex mechanisms underlying cell type-specific regulation.

## Conclusions

MAnorm exhibited excellent performance in quantitative comparison of ChIP-Seq data sets for both epigenetic modifications and transcription factors; the quantitative binding differences inferred by MAnorm were highly correlated with both the changes in expression of target genes and also the binding of cell type-specific regulators. With the accumulation of ChIP-seq data sets, MAnorm should serve as a powerful tool for obtaining a more comprehensive understanding of cell type-specific and cell state-specific regulation during organism development and disease onset.

## Materials and methods

The workflow of MAnorm is summarized in Figure 1. First, four bed files that describe the coordinates of all predefined peaks and aligned sequence reads of two ChIP-Seq samples are used as input. Second, MAnorm calculates the number of reads in a window of the same length centered at the summit of each peak. Here the window size should be comparable to the median length of ChIP-enriched regions; we recommend 2,000 bp window size for histone modifications and 1,000 bp for transcription factor binding sites. The (*M, A*) value of each peak is then defined as:

(1) $$M={\text{log}}_{2}\left(R_{1}/R_{2}\right)$$

and:

(2) $$A={\text{log}}_{2}\left(R_{1}\times R_{2}\right)/2$$

Here, *R_1 _*is the read density at this peak region in ChIP-Seq sample 1 and *R_2 _*is the corresponding read density in sample 2. To avoid log_2_0, we added a value of 1 to the real number of reads for all peaks. Thus, the value of *M *describes the log_2 _fold change of the read density at a peak region between two samples, while *A *represents the average signal intensity in terms of log_2_-transformed read density. To build the normalization model, each peak of the two samples being compared was further classified as a common or a unique peak, depending on whether or not it overlapped (by at least one nucleotide, as implemented in our analysis in this study) with any peak in the other sample. The downloadable MATLAB MAnorm package (Additional file 1) also provides a parameter for users to select common peaks based on a cutoff of peak summit-to-summit distance. By default, this value is set to 500 bp for histone modifications and 250 bp for transcription factors. In addition, when a peak overlaps with multiple peaks in the other sample, MAnorm selects the peak with the smallest summit-to-summit distance to avoid potential bias in building the normalization model. Next, robust regression was applied to the *M*-*A *values of common peaks using iterative re-weighted least squares with a bi-square weighting function [33] and a linear model was derived to fit the global dependence between the *M-A *values of these peaks:

(3) M=a+b×A

To normalize the (*M, A*) values of all peaks, MAnorm performed coordinate transformation to make the *A *axis overlap with the linear model derived from regression. The corresponding (*M, A*) value under the new coordinate system was then taken as the normalized (*M, A*) value of each peak. Finally, a *P*-value associated with each peak was calculated to quantify the significance of differential binding at this locus using a Bayesian model developed by Audic and Claverie [16]:

$$p\left(y|x\right)=\left(x+y\right)!/x!y!2^{x=y=1}$$

in which x and y specify the normalized read count at this peak in sample 1 and sample 2, respectively. Additional file 3 provides further details on *P*-value calculations. When the read densities at most peak regions are high, most peaks associated with absolute *M *values > 1 are associated with significant *P*-values. Then, the *M *value can be used to rank peaks and select differential binding regions, as was done in analyzing ENCODE ChIP-Seq data (Supplementary Table 1 in Additional file 4). When read densities at most peak regions are relatively low, some of the peaks associated with absolute *M *values > 1 may still fail to obtain significant *P*-values. In such a case, we suggest ranking peaks by *P*-values and defining differential binding regions using combined cutoffs of both *M *value and *P*-value, as we did when analyzing the ChIP-seq data from Taslim *et. al. *[12] (Supplementary Table 2 in Additional file 4).

The output of MAnorm includes the normalized (*M, A*) value and the corresponding *P*-value of each peak. To illustrate the normalization process, the (*M, A*) values of all peaks before and after normalization are plotted together with the linear model derived from common peaks. The MAnorm package will also generate three bed files presenting the genome coordinates for the non-differential binding region and two differential binding regions based on user-specified cutoffs, together with two wig files (corresponding to the two peak lists under comparison) that can be uploaded to a genome browser for visualization of the *M *value for each peak (Supplementary Figure 9). MATLAB and R versions of the MAnorm package are available for downloading in Additional file 1.

### Application of MAnorm to ENCODE ChIP-Seq data

The performance of MAnorm was tested using ENCODE ChIP-Seq data describing histone modifications (H3K4me3 and H3K27ac) [34] and transcription factor binding (c-Myc and Pol II) [35] across three human cell lines: H1 ES cells, HeLaS3 cells, and K562 cells [36]. Since these data were generated and processed by different laboratories associated with the ENCODE project, the data sets were reanalyzed and the ChIP-Seq peaks in each sample were redefined using MACS [4] using a *P*-value cutoff of 1e-10 for histone modifications and a *P*-value cutoff of 1e-6 for transcription factor binding. The peaks of histone modifications were further filtered by the false discovery rate (FDR) values modeled by MACS. The target genes of each group of peaks were defined as those RefSeq genes that have a given peak(s) in the promoter region, defined as the region from 8 kb upstream to 2 kb downstream of the transcription start site.

Gene expression data for all three cell types were collected from the Gene Expression Omnibus (GEO) database using accession numbers [GEO:GSE26312] (for H1 ES cells) [29], [GEO:GSE2735] (for HeLaS3 cells) [37] and [GEO:GSE12056] (for K562 cells) [38], and the raw data were reprocessed by dChip [39]. The differentially expressed genes were subsequently identified by Significance Analysis of Microarrays (SAM) [40] using a combined cutoff of fold change > 2 and FDR < 0.01. In total, 3,465 genes more highly expressed in H1 ES cells and 2,224 genes more highly expressed in K562 cells were identified from the H1 ES to K562 comparison; 5,815 genes more highly expressed in H1 ES cells and 1,649 genes more highly expressed in HeLaS3 cells were identified from the H1 ES cell to HeLaS3 cell comparison; and 3,555 genes more highly expressed in HeLaS3 cells and 5,916 genes more highly expressed in K562 cells were identified from the HeLaS3 cell to K562 cell comparison. To study the relationship between binding differences in peak regions and the expression change of the corresponding target genes, we used the *M *values of peaks to divide the targeted genes into different groups separated by integer *M *values from -4 to 4, and then calculated the enrichment score of the overlap between each gene group and those differentially expressed genes. To avoid extreme enrichment scores, groups composed of < 50 genes were merged with the larger of the adjacent two gene groups.

### Motif scan and hierarchical clustering of motif scores with peak *M *value

To detect the potential binding of transcription factors in defined peak regions, we downloaded the position weight matrixes of 130 core vertebrate motifs from the JASPAR database [41] and performed motif scan [42] applied to a 1,000 bp window centered at the peak summit. For each motif *F*, the raw motif matching score at each peak *P *was calculated as:

$$\underset{s\in p}{\text{max}}\left[\text{log}\frac{P\left(S|M\right)}{P\left(S|B\right)}\right]$$

in which *S *is a sequence fragment of the same length as the motif and *B *is the background frequency of different nucleotides estimated from 10,000 random 1,000 bp sequences sampled from the genome. The motif score of motif *M *in peak *P *was defined as the raw motif matching score divided by the maximum possible score, that is, the raw motif score obtained by the consensus sequence of the motif.

To identify transcription factors associated with cell type-specific binding of the ChIP'd proteins, we applied hierarchical clustering with Ward's linkage to cluster the *M *value with the motif matching score of JASPAR motifs in all peaks of cell type 1, and separately the -*M *value was clustered with the motif scores in all peaks of cell type 2, using '1 - *ρ*' as the distance metric, where *ρ *is the Pearson correlation coefficient. Only motifs with an enrichment score > 1.2 and Bonferroni-corrected *P*-value < 1.0E-5 by Fisher exact test are shown in the clustering plots.

### Comparing the performance of MAnorm and other methods

For total read normalization, we divided the read intensity of each peak region by the total number of mapped sequence reads. For quantile normalization, we first divided the whole genome into non-overlapping bins of the same size as the window used in MAnorm (2,000 bp for H3K27ac), and then calculated the read count in each bin. Finally, the distribution of bin read counts was normalized to be the same by matching all quantiles between samples. For normalization by genome-wide MA plots, we first divided the whole genome into non-overlapping bins of the same size as the window used in MAnorm (2,000 bp for H3K27ac), and then calculated the *M-A *value of each bin. The dependence between *M*-*A *value was then removed by subtracting *M *values with local linear model fitted by LOWESS regression from the genome-wide *M*-*A *values.

To compare the performance of MAnorm with the model developed by Taslim *et al. *[12], we used MACS to identify peaks from the same Pol II ChIP-Seq datasets used by [12], and then applied MAnorm to compare Pol II binding profiles between estradiol (E2)-stimulated MCF7 cells and unstimulated MCF7 cells. The gene expression data of unstimulated and E2-stimulated MCF7 cells was obtained from the GEO database, accession number [GEO:GSE11352] [43]. We identified 59 genes showing higher expression in unstimulated MCF7 cells and 130 genes showing higher expression in E2-stimulated (12 h) MCF7 cells using SAM with fold change > 2 and FDR < 0.1. Finally, the performance of MAnorm was evaluated by comparing the difference of Pol II binding determined by both models with the differential expression of target genes.

## Abbreviations

ChIP-Seq: chromatin immunoprecipitation followed by massively parallel DNA sequencing; E2: estradiol; ES: embryonic stem; FDR: false discovery rate; GEO: Gene Expression Omnibus; S/N: signal to background noise.

## Competing interests

The authors declare that they have no competing interests.

## Authors' contributions

ZS and YZ conceived the study, developed the algorithms, carried out analyses and drafted the manuscript; DJW and SHO conceived the study, supervised the data analyses and edited the manuscript. All authors discussed the results and revised the manuscript.

## Supplementary Material

Additional file 1 **MAnorm package written in MATLAB and R**. Click here for file

Additional file 2 **Supplementary figures**. Click here for file

Additional file 3 **Supplementary text**. Includes text on the use of MAnorm normalized read density to determine whether the peak calling cutoff is comparable between two ChIP-seq data sets; results of downstream analyses following MAnorm are robust to different peak cutoffs; integrating multiple replicates in ChIP-seq data set comparison; derivation of the *P*-value that quantifies the significance of differential binding at peak regions; using MAnorm to compare H3K36me3 ChIP-seq data; assessing the effect of number of common peaks used in analysis; comparing signal-to-noise ratio before and after normalization; Supplementary Methods. Click here for file

Additional file 4 **Supplementary Tables - comparison of MAnorm with other methods**. Click here for file
