# Supplementary material for: MAnorm: a robust model for quantitative comparison of ChIP-Seq data sets
Source: Genome Biol. 2012 Mar 16;13(3):R16. doi: 10.1186/gb-2012-13-3-r16 (PMC3439967; doi:10.1186/gb-2012-13-3-r16)
Supplement: Additional file 2 — Supplementary figures. [file gb-2012-13-3-r16-S2.PDF]

Supplementary Figure 1

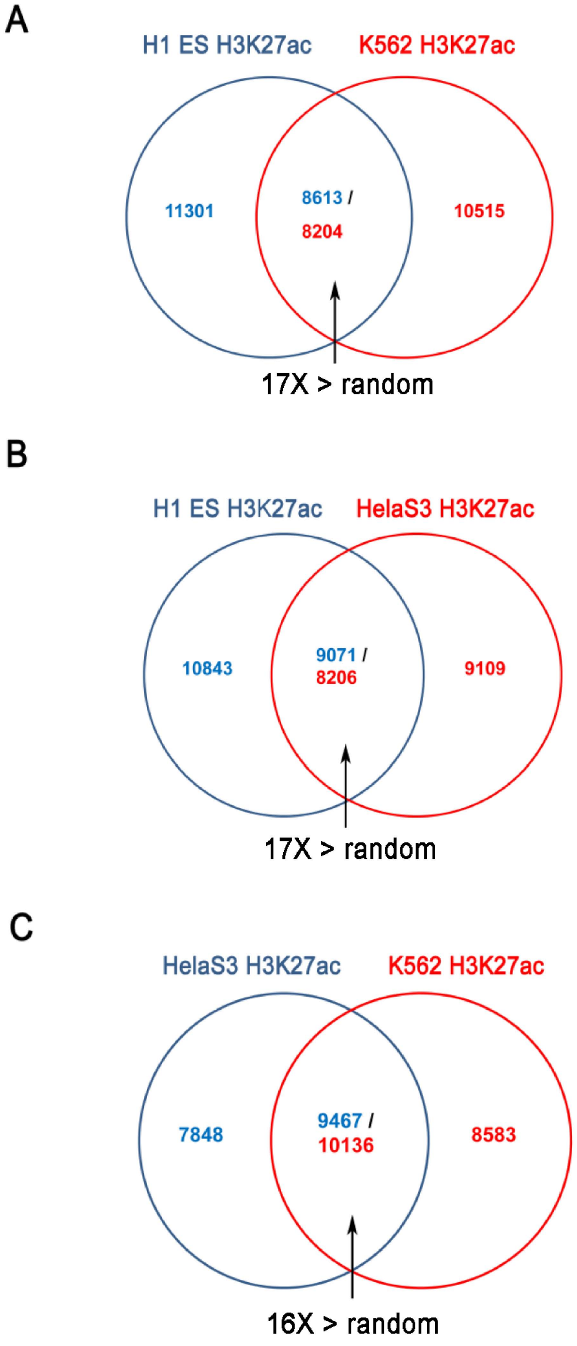

Supplementary Figure 1.Overlap of H3K27ac peaks in H1 ES, K562 and HelaS3 cells.
(A) Venn diagram depicting the overlap of H3K27ac peaks in H1 ES cells and K562 cells. The fold-change under each diagram is based on a comparison to the overlap of permutated peaks.
(B) Venn diagram depicting the overlap of H3K27ac peaks in H1 ES cells and HelaS3 cells.
(C) Venn diagram depicting the overlap of H3K27ac peaks in H1 HelaS3 cells and K562 cells.

Supplementary Figure 2

A

Before rescale

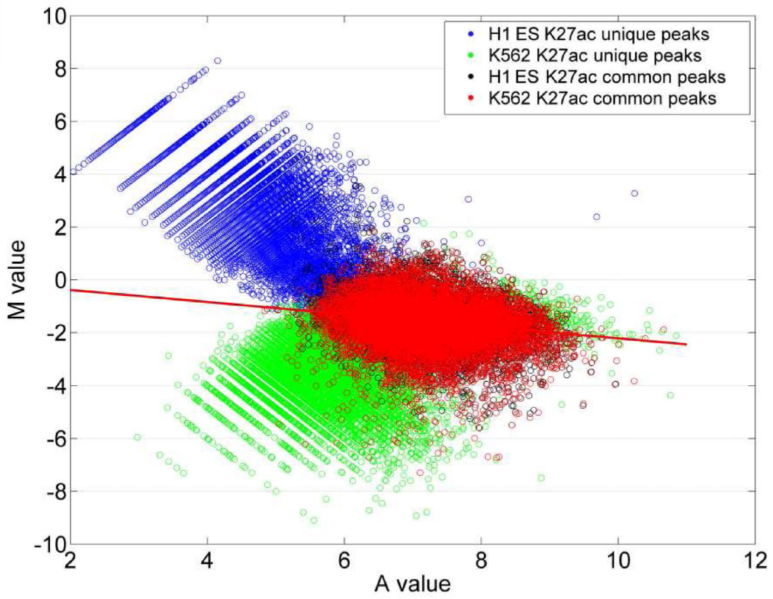

After rescale

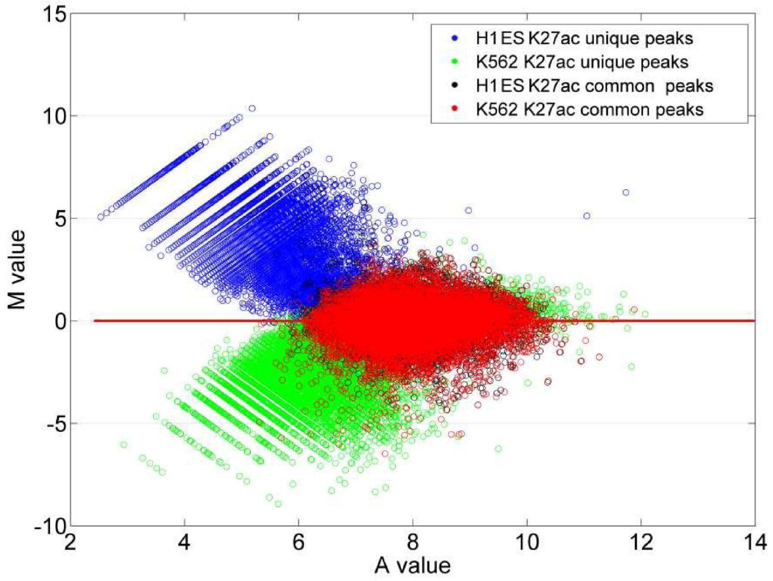

$-\log_{10}$(P-value)
after rescale

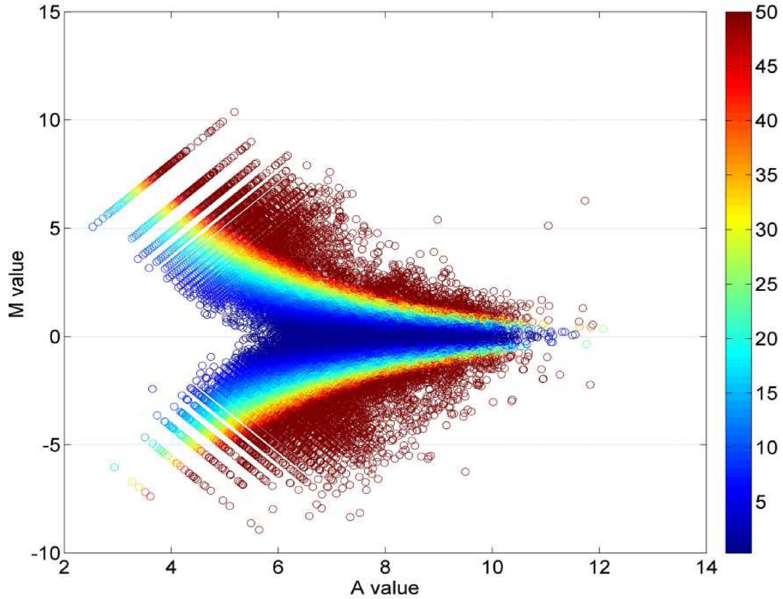

B

Before rescale

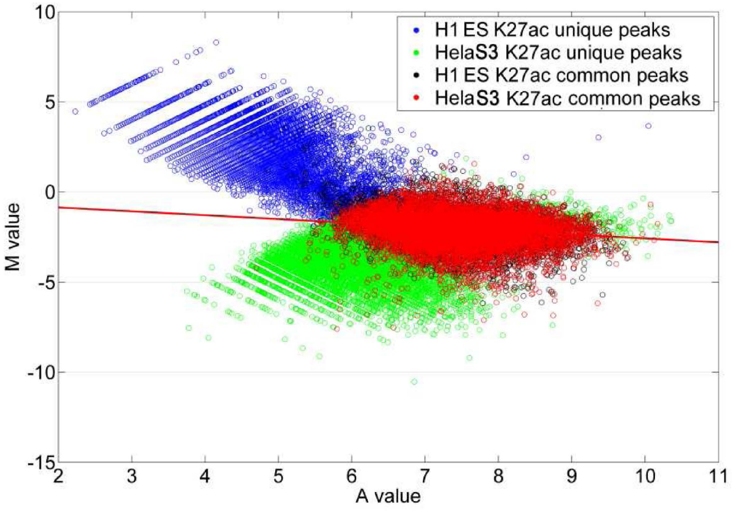

After rescale

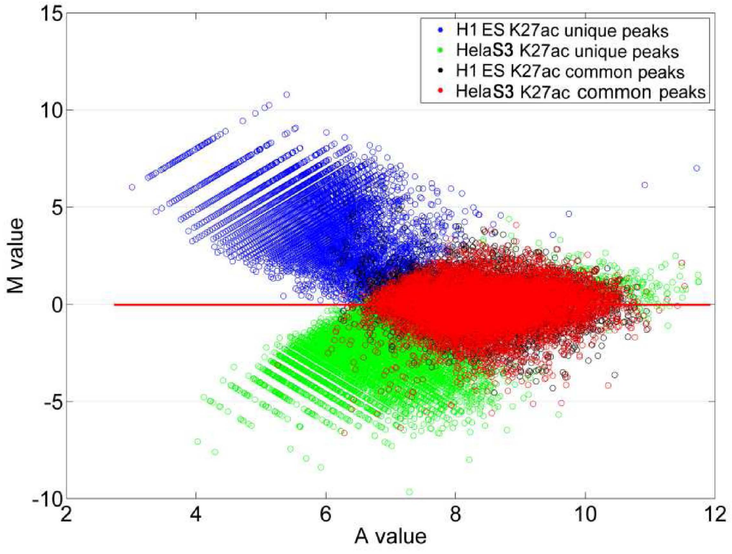

-log$_{10}$(P-value) after rescale

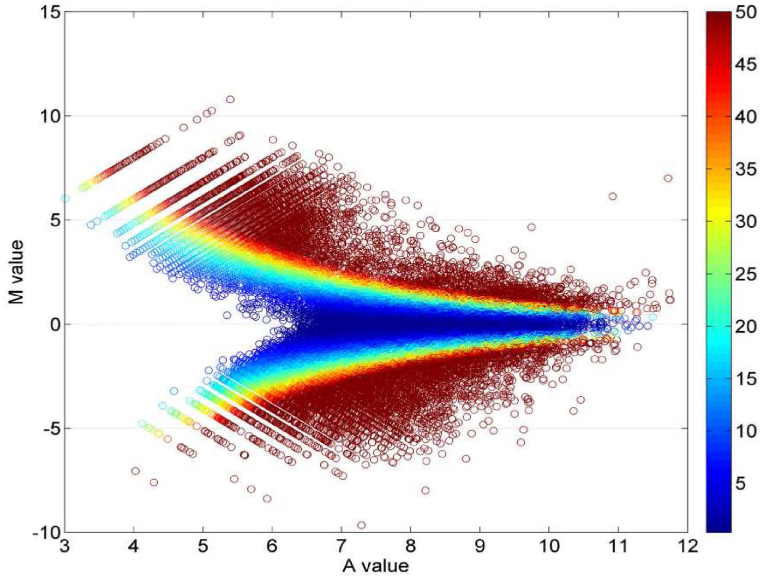

C

Before rescale

After rescale

-log$_{10}$(P-value) after rescale

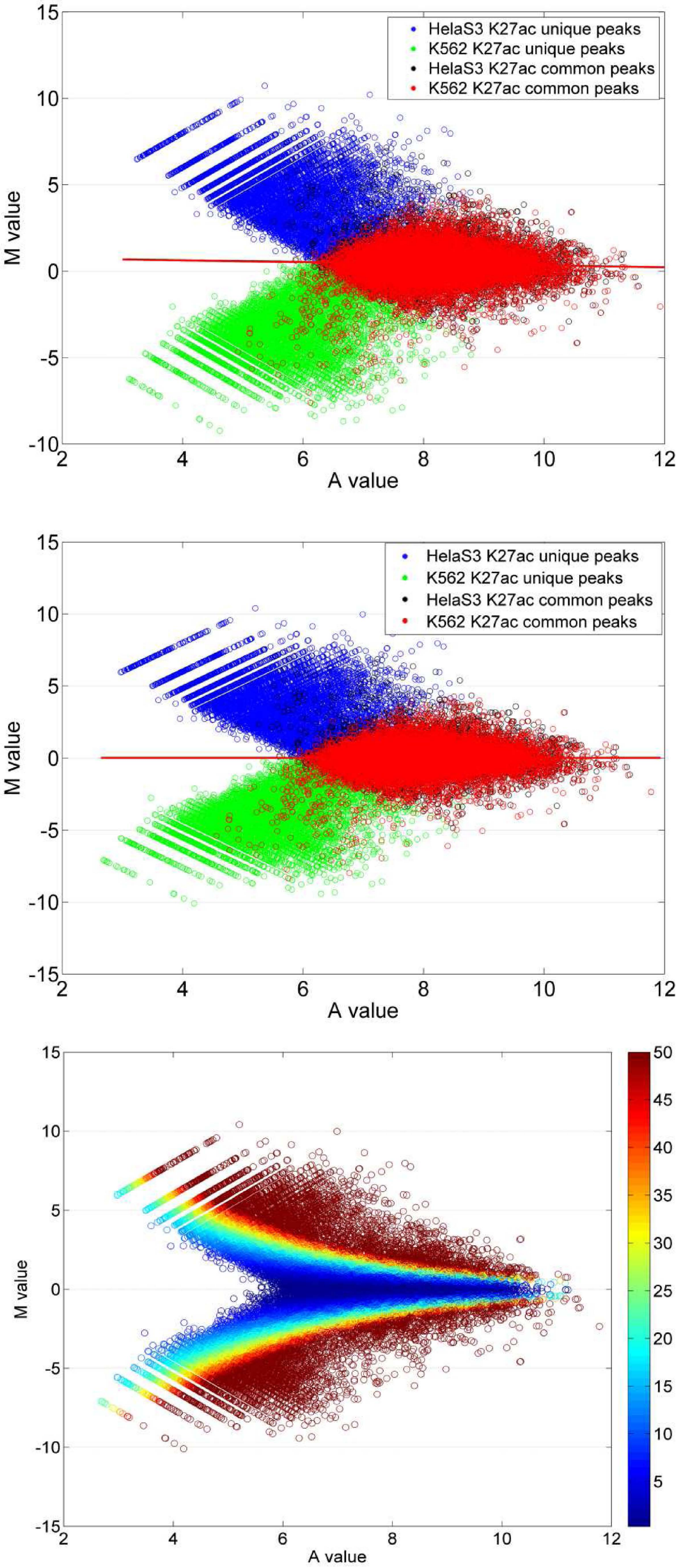

Supplementary Figure 2. MA plots comparing H3K27ac marks in H1 ES, K562 and HelaS3 cells before and after MAnorm, and p-values indicating significance of differential binding.

(A) MA plot of H3K27ac marks between H1 ES and K562 cells before and after MAnorm.

(B) MA plot of H3K27ac marks between H1 ES and HelaS3 cells before and after MAnorm.

(C) MA plot of H3K27ac marks between HelaS3 and K562 cells before and after MAnorm.

Red line is the linear model derived from common peaks by robust regression. P-values indicating the significance of binding difference between samples after MAnorm are represented by the color scale, with the color range on the right color bar indicating the –log10(p-value).

Supplementary Figure 3

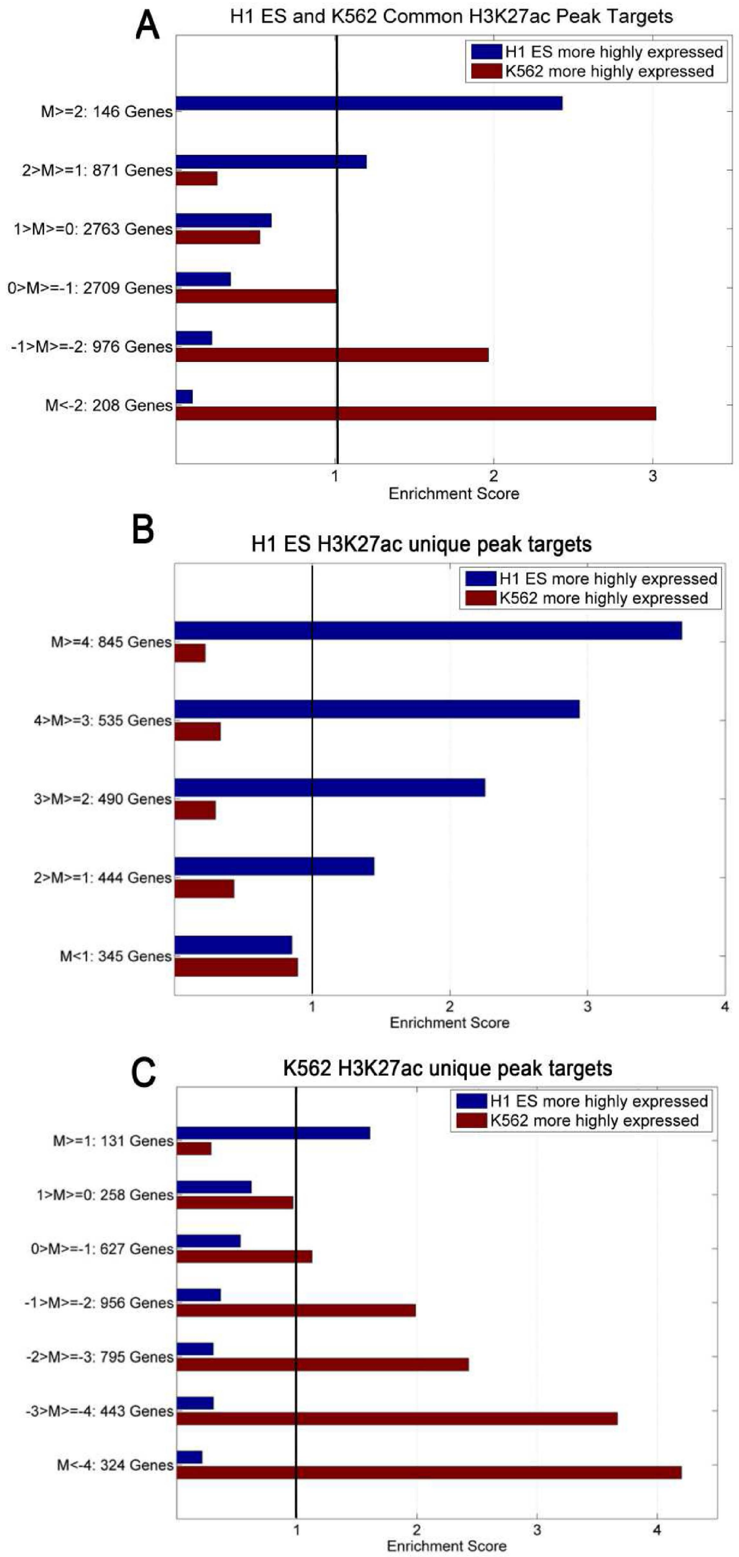

Supplementary Figure 3. Cell type-specific H3K27ac binding correlates well with cell type-specific expression of H3K27ac peak targets.

(A) Enrichment of the target genes of all common H3K27ac peaks in H1 ES cells and K562 cells in cell-type specifically expressed genes, as identified by SAM.

(B-C) Enrichment of the target genes of all unique H3K27ac peaks in H1 ES cells (B) or K562 cells (C) in cell-type specifically expressed genes.

Supplementary Figure 4

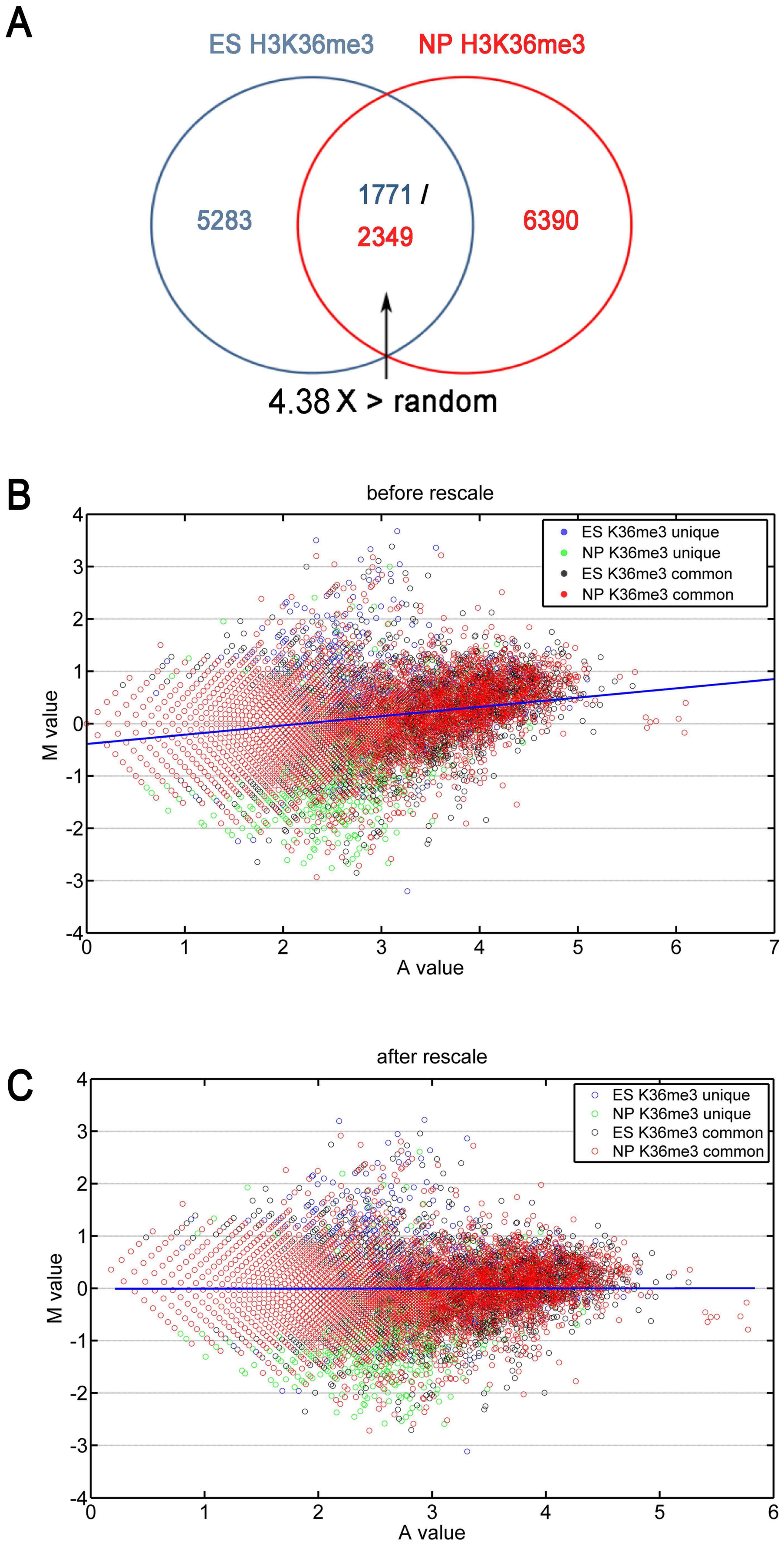

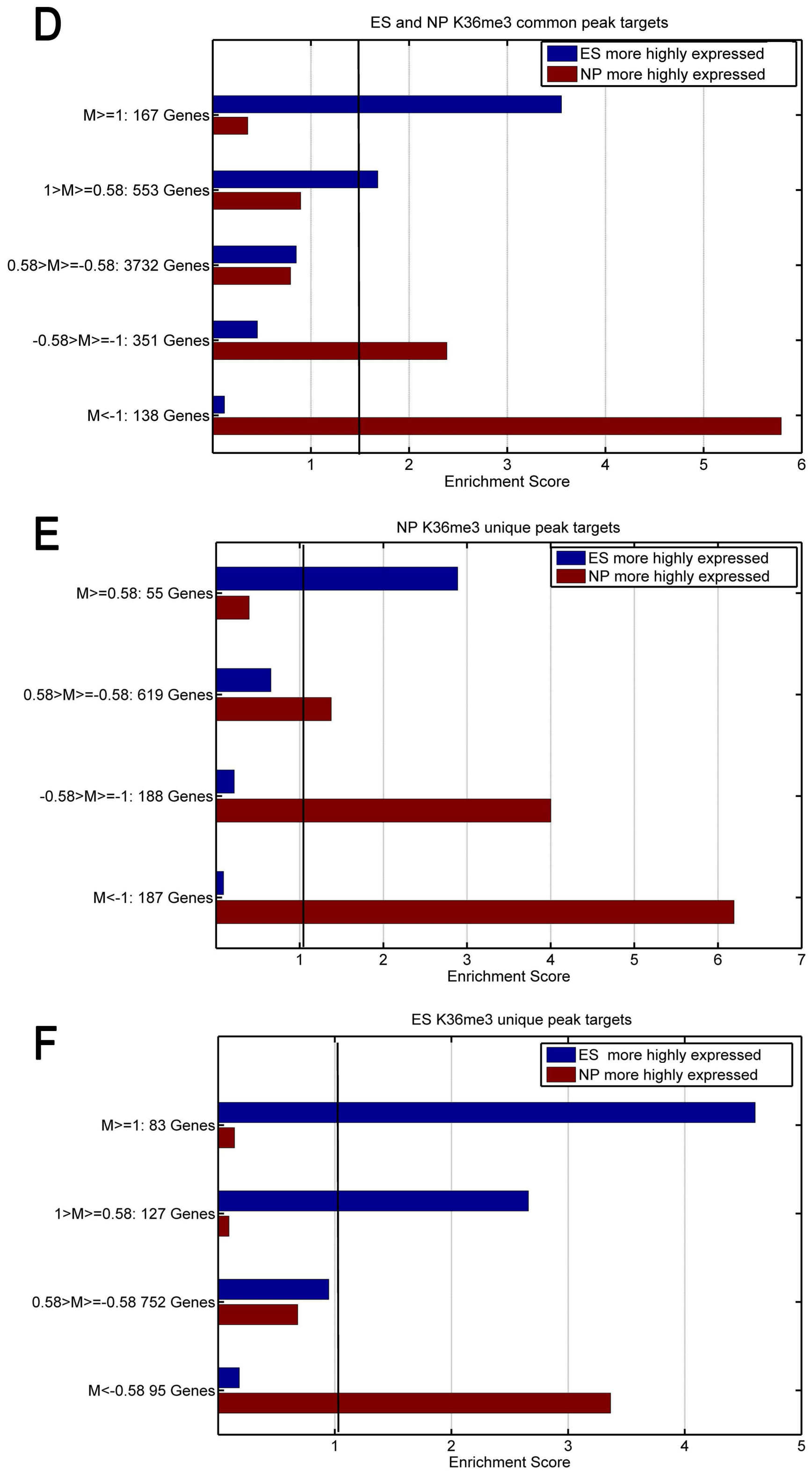

Supplementary Figure 4. Using MAnorm to quantitatively compare broad domains of histone modifications.
(A) Venn diagram depicting the overlap of H3K36me3 domains in mouse ES and NP cell lines. The fold-change under the diagram is based on a comparison to the overlap of permutated peaks.
(B-C) MA plot of H3K36me3 domains between mouse ES and NP cell lines before (B) and after (C) MAnorm.
(D-F) Enrichment of common (D) and unique (E, F) H3K36me3 domain targets grouped by M values in genes differentially expressed between ES and NP cells.

Supplementary Figure 5

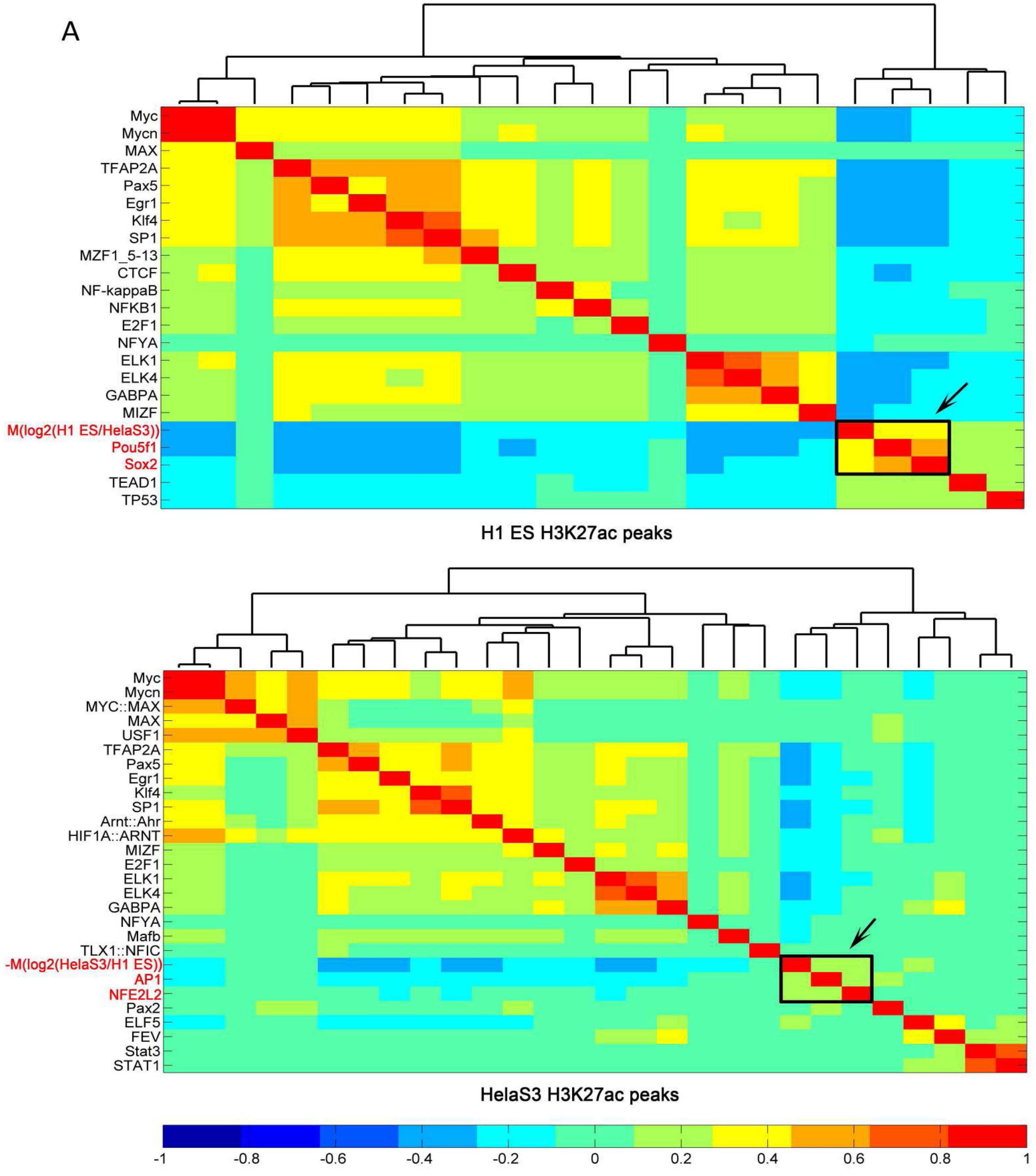

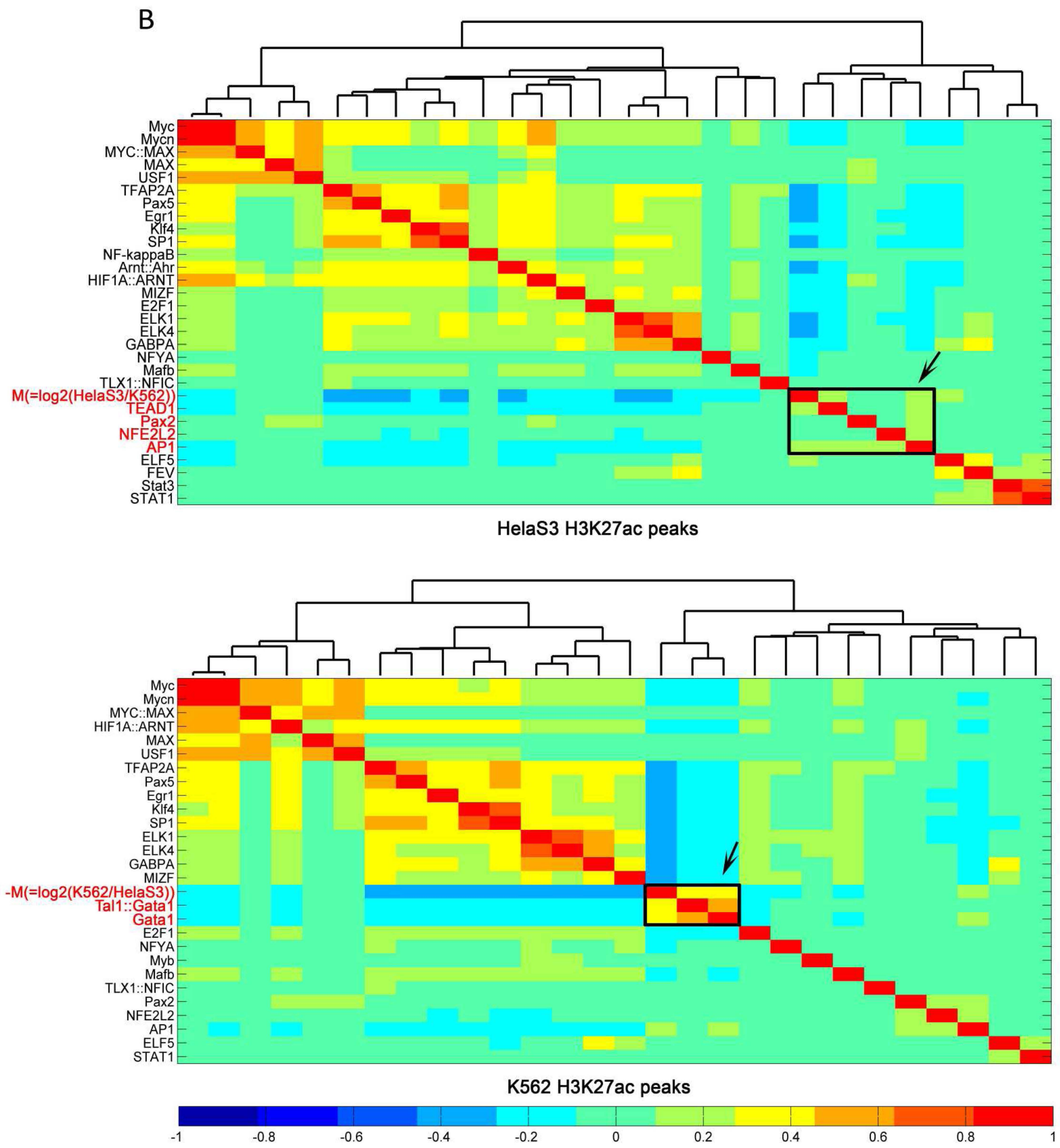

Supplementary Figure 5.Hierarchical clustering of correlation coefficients between motif scores and *M* values of H3K27ac peaks for comparison between H1 ES and HelaS3 cells (A) or between HelaS3 and K562 cells (B).

Supplementary Figure 6

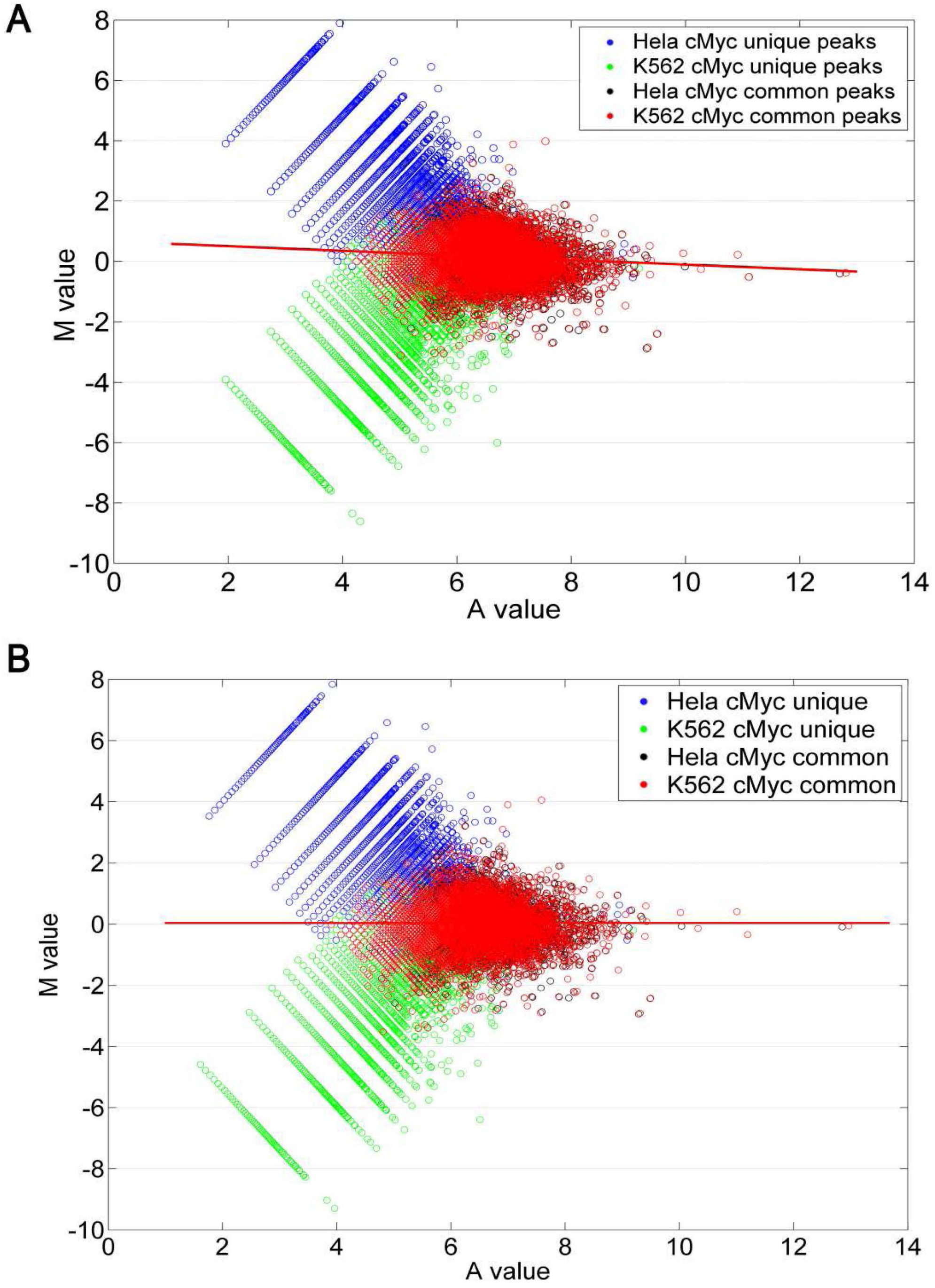

Supplementary Figure 6. MA plots comparing cMyc binding in HelaS3 and K562 cells before (A) and after (B) MAnorm. Red line in (A) is the linear model derived from common peaks by robust regression.

Supplementary Figure 7

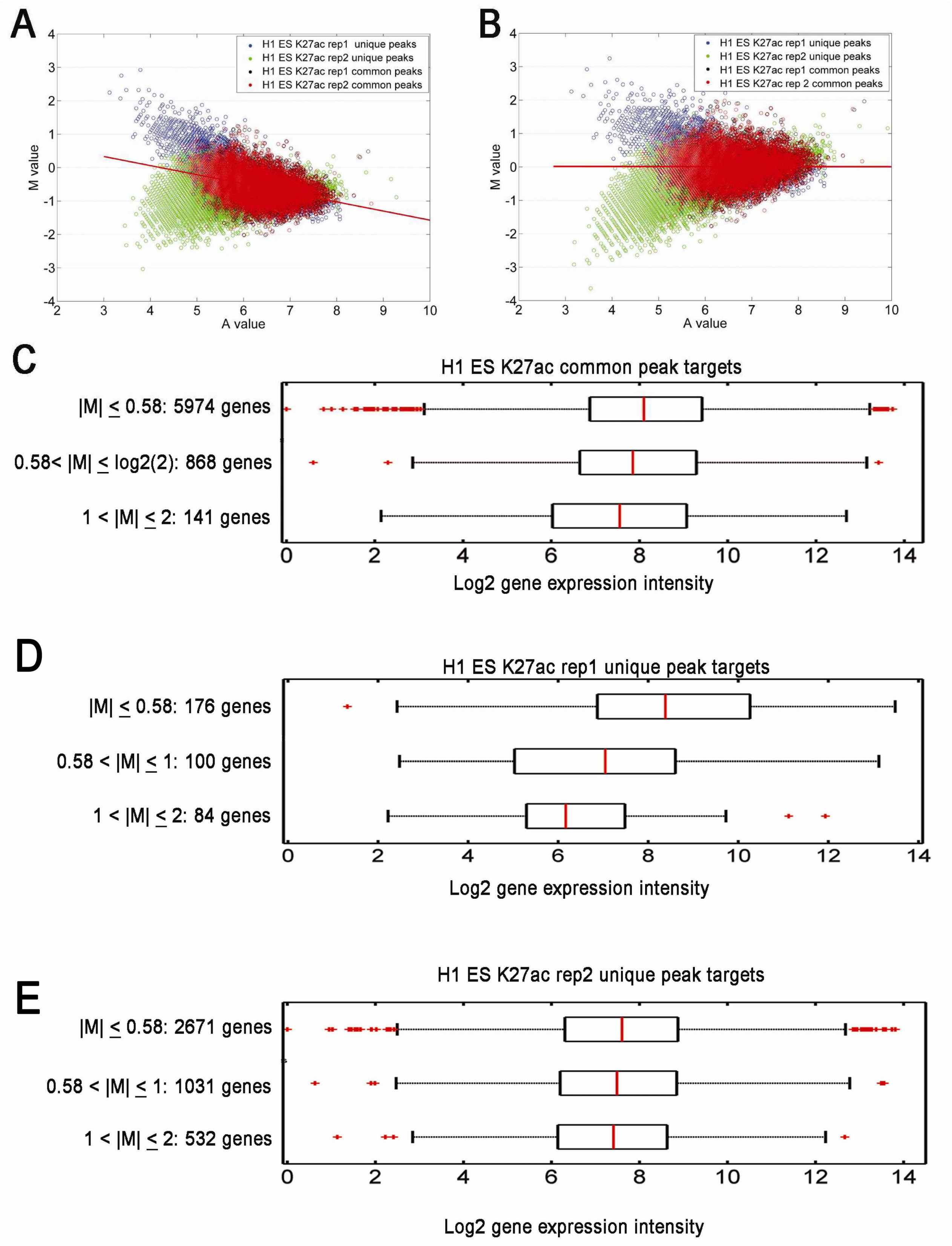

F

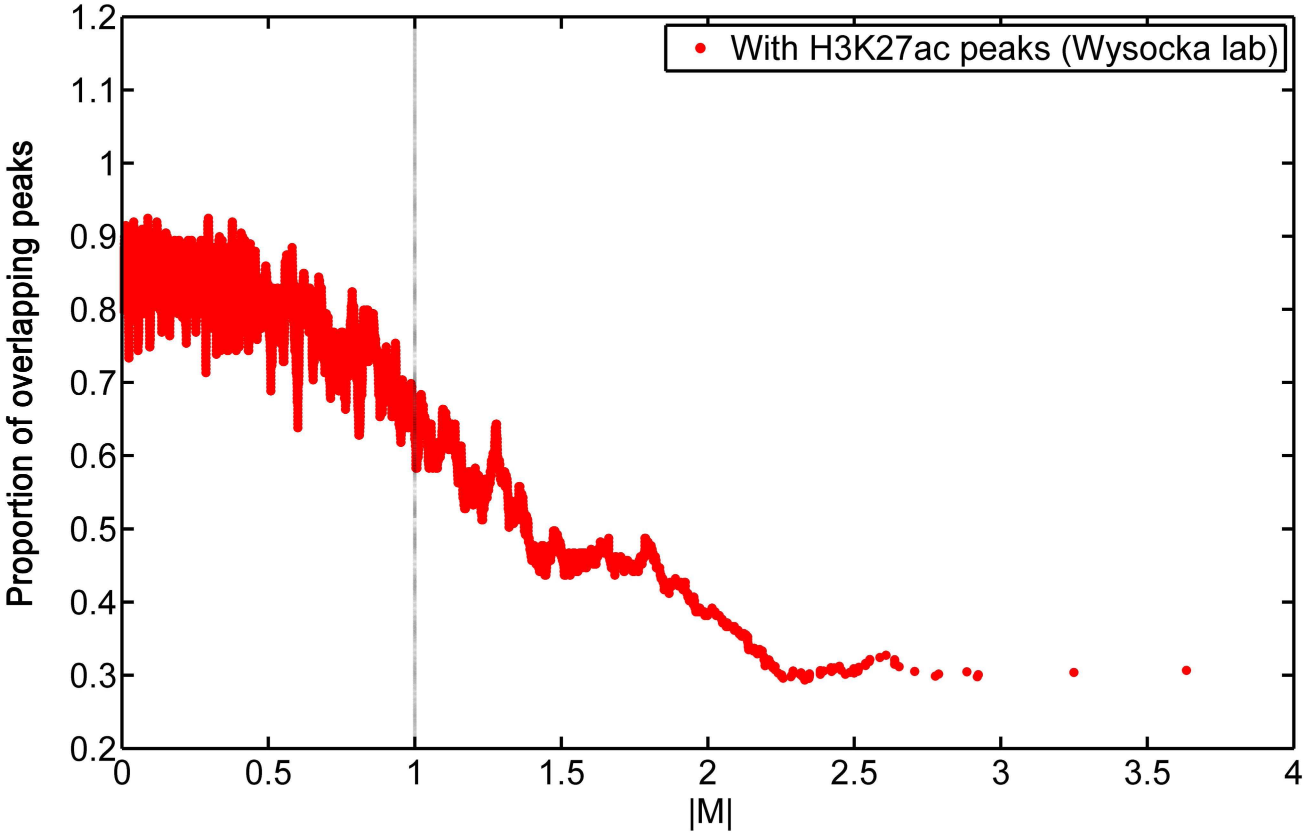

Supplementary Figure 7. Application of MAnorm to the integration of ChIP-Seq replicates.
(A-B) MA plot comparing H3K27ac marks between two H1 ES replicates before (A) and after (B) MAnorm.
(C-E) Common (C) and unique (D-E) H3K27ac peak targets between two H1 ES replicates were divided to 3 groups based on absolute M value. The box-plots show log2 gene expression values for the genes in each group.
(F) The fraction of ENCODE H3K27ac peaks that overlap with H3K27ac peaks in H1 ES cells based on data fromRadaIglesias et al [19]. Peaks were ranked from high to low |M| values, and the proportion of overlap was calculated using a moving window of 200 peaks.

Supplementary Figure 8

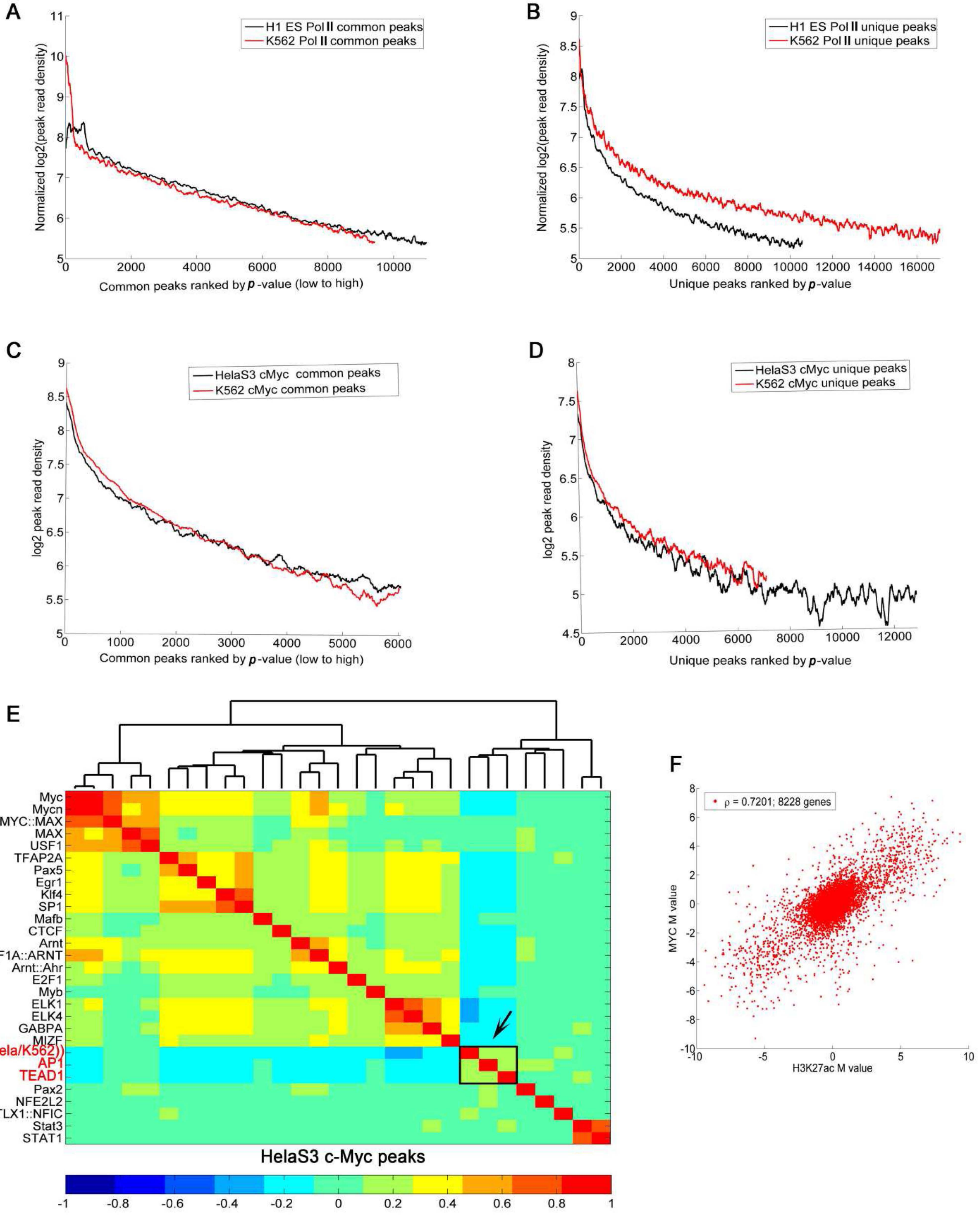

Supplementary Figure 8. Evaluation of p-value cutoff in peak calling.

(A-B) normalized read intensities versus p-values of Pol II peaks in H1 ES and K562 common peaks (A) and unique peaks (B). X-axis represents the common peaks (A) or unique peaks (B) ranked from low to high MACS p-values. Y-axis displays the log2 read intensity normalized by MAnorm.

(C-D) normalized read densities versus p-values for cMyc common peaks (C) and for cMyc unique peaks (D) in HelaS3 and K562 cells.

(E) Hierarchical clustering of motif scores and –M value of HelaS3 cMyc peaks after filtering lower read intensity peaks as compared to K562 peaks. Only the significantly enriched motifs are shown.

(F) Scatter plot of M valuesfor c-Myc comparison, after filtering lower read intensity peaks as compared to K562 peaks, versus M valuesfor H3K27ac comparison between HelaS3 and K562 cells.

Supplementary Figure 9

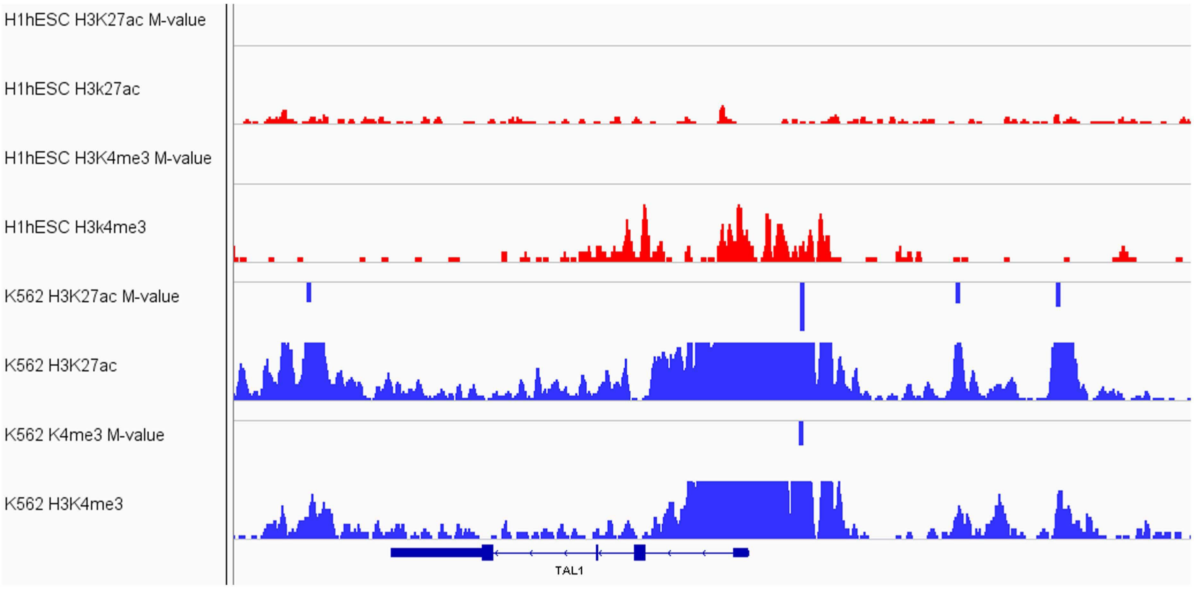

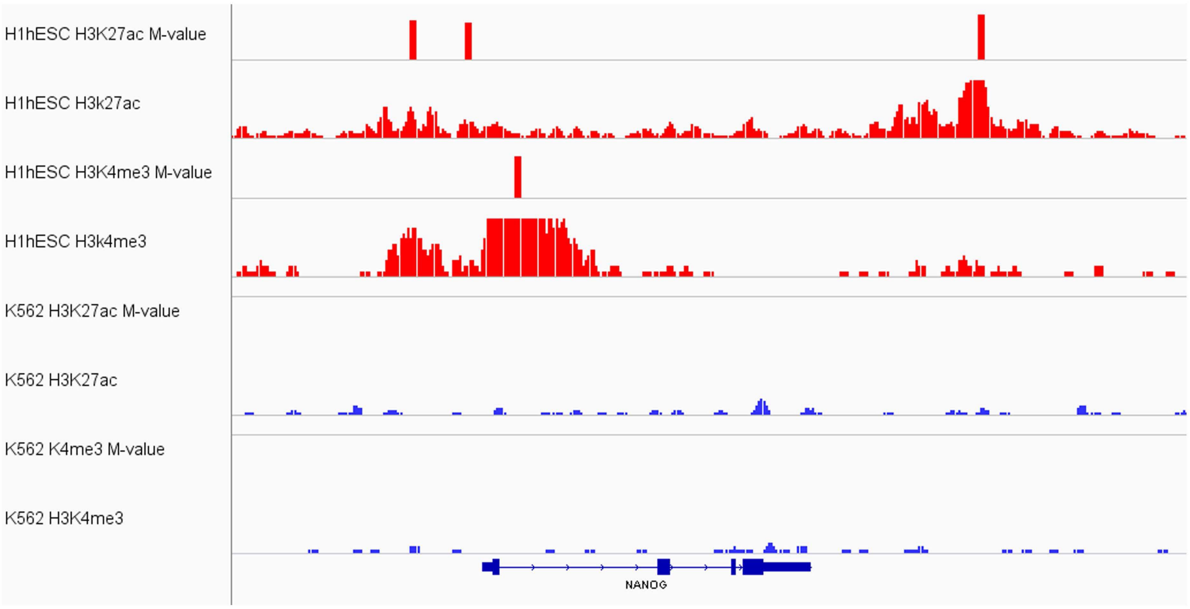

Supplementary Figure 9.Integrative Genomics Viewer (IGV) screen shots of *M* values for peaks around TAL1 (SCL) and NANOG genes.

The position of each bar in the *M* value track marks the region100 bp up and down from the peak summit, and the height of the bar correlates with the peak's *M* value. Users can also use the color view mode in IGV to distinguish between positive and negative *M* values.

Supplementary Figure 10

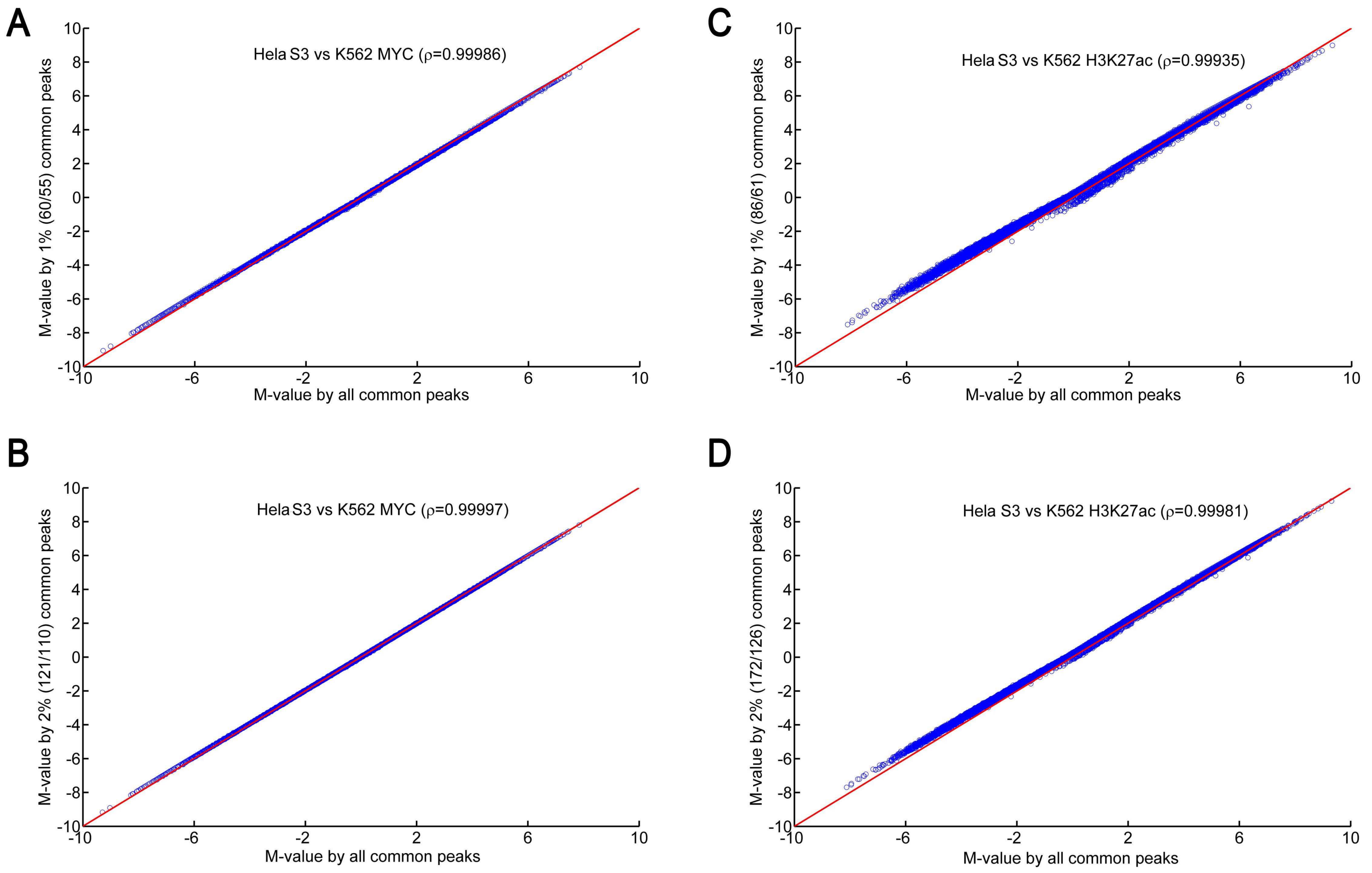

Supplementary Figure 10. High correlation of *M* values inferred from all common peaks and from a subsetof common peaks.

(A-B) Correlation of *M* values of c-Myc peaks between HelaS3 and K562 cells calculated based only on 1% (A) or 2% (B) of common peaks versus M values obtained based on all common peaks.

(C-D) Correlation of *M* values of H3K27ac peaks between H1 ES and K562 cells calculated based only on 1% (C) or 2% (D) of common peaks versusM values obtained based on all common peaks.
