# Supplementary material for: MAnorm: a robust model for quantitative comparison of ChIP-Seq data sets
Source: Genome Biol. 2012 Mar 16;13(3):R16. doi: 10.1186/gb-2012-13-3-r16 (PMC3439967; doi:10.1186/gb-2012-13-3-r16)
Supplement: Additional file 3 — Supplementary text. Includes text on the use of MAnorm normalized read density to determine whether the peak calling cutoff is comparable between two ChIP-seq data sets; results of downstream analyses following MAnorm are robust to different peak cutoffs; integrating multiple replicates in ChIP-seq data set comparison; derivation of the P-value that quantifies the significance of differential binding at peak regions; using MAnorm to compare H3K36me3 ChIP-seq data; assessing the effect of number of common peaks used in analysis; comparing signal-to-noise ratio before and after normalization; Supplementary Methods. [file gb-2012-13-3-r16-S3.DOC]

**Supplementary Text**

**Use of MAnorm normalized read density to determine whether the peak calling cutoff is comparable between two ChIP-seq data sets -** Most peak calling programs use the information from background regions to model the significance of peak candidates, which often are not equivalent between samples when the signal to background noise ratio varies across samples. Consequently, selecting a comparable cutoff to define peaks is important for ChIP-Seq data comparison. From the definition of *M* and *A*, it is apparent that the normalized read density in each peak region can be calculated from the normalized *(M, A)* values (see Supplementary Methods). In cases where the cutoff p-value is comparable between two samples, peaks close to the cutoff can be expected to have similar normalized read densities between samples, especially when the peak sets are identified by the same peak-calling program. Based on this idea, we used a simple method to visually determine whether the cutoffs used to define peaks in two ChIP-Seq data sets are comparable to each other. This idea is illustrated in Supplementary Fig. 8A, 8B using Pol II ChIP-Seq data sets in H1 ES and K562 cells. Applying the same cutoff to both cell lines (see Methods), 21,570 Pol II peaks were identified in H1 ES cells and 26,519 Pol II peaks were identified in K562 cells using MACS software. ~11,000 of these peaks were common to both peak sets. Next, both the common and the unique peaks within each cell line’s peak set were ranked by the *p*-value given by MACS, and then the normalized read densities were plotted separately for visual comparison for both the common and the unique peaks (Supplementary Fig. 8A, 8B). It is apparent that the normalized read density of K562 unique peaks of Pol II drops much more slowly with increasing MACS *p*-value as compared to the normalized read density of the H1 ES unique peaks (Supplementary Fig. 8B). Moreover, peaks close to the cutoff of each cell line had similar normalized read densities (i.e., Y-axis values of ~5.2-5.3 in both cases), despite the fact that many more unique peaks were identified in K562 cells (17,095 peaks) as compared to H1 ES cells (10,560 peaks). Thus the cutoffs of peak calling for Pol II in this example are comparable with respect to peak density between the two data sets. However, when this method is applied to the cMyc peaks in HelaS3 and K562 cells, the normalized read density drops in parallel for both cell lines as the *p*-value increases (Supplementary Fig. 8D). This suggests the much larger number of c-Myc peaks identified in HelaS3 cells might not be reasonable, and thus the cutoff values used might not be comparable. Based on results such as these, the user may apply a more stringent cutoff value to the HelaS3 c-Myc data set, as indicated below, to make the two sets of peaks more comparable.

**Results of downstream analyses following MAnorm are robust to different peak cutoffs -** To test whether analyses based on MAnorm are impacted by the cutoff used for peak calling, we removed the higher p-value peaks from the HelaS3 c-Myc peak list, such that the total number of HelaS3 cell and K562 cell c-Myc peaks was the same (13140 peaks). Downstream analyses carried out using the modified set of peaks showed that the hierarchical clustering (Supplementary Fig. 8E) and the correlation between the H3K27ac *M* values and c-Myc *M* values (Supplementary Fig. 8F) were generally unchanged (compared with Fig. 5C, 5D), indicating MAnorm is relatively robust to the cutoff value used for peak calling.

**Integrating multiple replicates in ChIP-seq data set comparison -** We tested whether using multiple replicates in ChIP-seq data sets compared by MAnorm could improve the performance. We collected H3K27ac ENCODE ChIP-Seq data from both H1 ES cells and K562 cells, each with two replicates, and applied MAnorm to perform comparisons between two cell types based on all four possible combinations. By using the averaged *M* value from these four comparisons, the final *M* values showed slightly improved performance in covering differentially expressed genes, suggesting they captured more comprehensive information of ChIP-seq signal changes (Supplementary Table 3).

**Derivation of the p-value that quantifies the significance of differential binding at peak regions -** From Audic and Claverie , we can infer the conditional probability of observing *y* reads in a region from sample 2 with *N_2_* total reads, given *x* reads in the same region in sample 1 with *N_1_* total reads:

*p(y|x) =(N_2_/N_1_)* (*x+y*)! */* [*x!y!*(1+ *N_2_/N_1_*)*^x+y+1^*]

Since after normalization, the common peaks have almost the same number of total reads between two samples, this conditional probability could be simplified to

*p(y|x) = (x+y)!/x!y!2^x+y+1^*

, which can then be applied to all peaks.

**Using MAnorm to compare H3K36me3 ChIP-seq data -** MAnorm was applied to compare H3K36me3 ChIP-Seq data sets between mouse ES and NP cells . We downloaded the broad domains of H3K36me3 modification in these two cells defined by the RSEG software package , and then calculated the normalized *M-A* values of these domains based on a window of size 6 kb. By comparing the expression levels of the genes in which the domains are located, we found the *M* values of H3K36me3 domains are highly correlated with the expression changes of target genes (Supplementary Fig. 4), indicating MAnorm can be used to compare ChIP-seq data for broad, diffuse histone modifications. We also found when applying MAnorm to compare H3K36eme3 domains that the model showed good performance despite the amount of peak overlap being only approximately 4-fold more than random permutation (compared to 16~65-fold for H3K4me3 and H3K27ac).

**Assessing the effect of number of common peaks used in analysis -** MAnorm relies on the assumption that chromatin-associated protein binding at the common peaks of two samples are determined by similar mechanisms, thus the scaling relationship of observed ChIP-seq signals inferred from these peaks can be used to normalize the signal at all peaks. In general, the higher the enrichment observed for the overlap between two peak sets, the more likely this assumption would be true.

We investigated the number of common peaks needed to build a reliable model for normalization. We randomly selected 1% or 2% of common peaks and re-calculated the *M* value for all peaks based on the *M-A* dependency inferred from these small sets of common peaks. We found that *M* values inferred from the subsets of common peaks were highly similar to those inferred from all common peaks (Supplementary Fig. 10), indicating MAnorm is not sensitive to the absolute number of common peaks used in the analysis.

**Comparing the signal-to-noise ratio before and after normalization -** We compared the signal-to-noise ratios (S/N) before and after normalization, as follows. We defined the S/N ratio as (number of reads in peaks) / (number of reads outside peaks), for the H3K27ac mark for H1 ES and K562. For the S/N ratio after normalization, we defined it as (number of reads in peaks normalized by MAnorm) / (number of reads outside peaks normalized by total number of reads). We found the S/N ratios for the two cell types were quite different (0.12 for H1 ES cells vs. 0.52 for K562 cells) before normalization, but they became much more similar (0.48 for H1 ES cells vs. 0.53 for K562 cells) after MAnorm, indicating MAnorm can be used to analyze samples with strong S/N differences.

**Supplementary Methods**

**Cutoff Diagnostic of peak calling -** To determine whether a given cutoff was reasonable for ChIP-Seq data comparison, we calculated the normalized read densities of each peak in both ChIP-Seq data sets (R1* and R2*) as follows:

log_2_(R_1_*) = log_2_(R_1_)

*log_2_(R_2_*) = 2*a/(2-b)+(2+b)log_2_(R_2_)/(2-b)*

, in which the values of coefficients *a* and *b* were obtained from the linear model (equation 3) derived from common peaks.

**Supplementary References**

1. Audic S, Claverie JM: **The significance of digital gene expression profiles.** *Genome Res* 1997, **7:**986-995.

2. Mikkelsen TS, Ku M, Jaffe DB, Issac B, Lieberman E, Giannoukos G, Alvarez P, Brockman W, Kim TK, Koche RP, et al: **Genome-wide maps of chromatin state in pluripotent and lineage-committed cells.** *Nature* 2007, **448:**553-560.

3. Song Q, Smith AD: **Identifying dispersed epigenomic domains from ChIP-Seq data.** *Bioinformatics* 2011, **27:**870-871.
